# A mouse model for a partially inactive obesity-associated human *MC3R* variant

**DOI:** 10.1038/ncomms10522

**Published:** 2016-01-28

**Authors:** Bonggi Lee, Jashin Koo, Joo Yun Jun, Oksana Gavrilova, Yongjun Lee, Arnold Y. Seo, Dezmond C. Taylor-Douglas, Diane C. Adler-Wailes, Faye Chen, Ryan Gardner, Dimitri Koutzoumis, Roya Sherafat Kazemzadeh, Robin B. Roberson, Jack A. Yanovski

**Affiliations:** 1 Section on Growth and Obesity, Program in Developmental Endocrinology and Genetics, NICHD, National Institutes of Health, 10 Center Drive, Bethesda, Maryland 20892, USA; 2 Mouse Metabolism Core Laboratory, NIDDK, National Institutes of Health, 10 Center Drive, Bethesda, Maryland 20892, USA; 3 Heritable Disorders Branch, NICHD, National Institutes of Health, 10 Center Drive, Bethesda, Maryland 20892, USA; 4 Cell Biology and Metabolism Program, NICHD, National Institutes of Health, 10 Center Drive, Bethesda, Maryland 20892, USA

## Abstract

We previously reported children homozygous for two *MC3R* sequence variants (C17A+G241A) have greater fat mass than controls. Here we show, using homozygous knock-in mouse models in which we replace murine *Mc3r* with wild-type human (*MC3R*^*hWT/hWT*^) and double-mutant (C17A+G241A) human (*MC3R*^*hDM/hDM*^) *MC3R*, that *MC3R*^*hDM/hDM*^ have greater weight and fat mass, increased energy intake and feeding efficiency, but reduced length and fat-free mass compared with *MC3R*^*hWT/hWT*^. *MC3R*^*hDM/hDM*^ mice do not have increased adipose tissue inflammatory cell infiltration or greater expression of inflammatory markers despite their greater fat mass. Serum adiponectin levels are increased in *MC3R*^*hDM/hDM*^ mice and *MC3R*^*hDM/hDM*^ human subjects. *MC3R*^*hDM/hDM*^ bone- and adipose tissue-derived mesenchymal stem cells (MSCs) differentiate into adipocytes that accumulate more triglyceride than *MC3R*^*hWT/hWT*^ MSCs. *MC3R*^*hDM/hDM*^ impacts nutrient partitioning to generate increased adipose tissue that appears metabolically healthy. These data confirm the importance of MC3R signalling in human metabolism and suggest a previously-unrecognized role for the MC3R in adipose tissue development.

## Abstract (web summary)

The melanocortin receptor, MC3R, regulates organismal energy homeostasis. Here, Lee *et al*. create knock-in mice with the a mutated version of the human MC3R receptor found in obese children, and show these mice have more fat and smaller bone, yet are by and large metabolically healthy.

## Graphical abstract

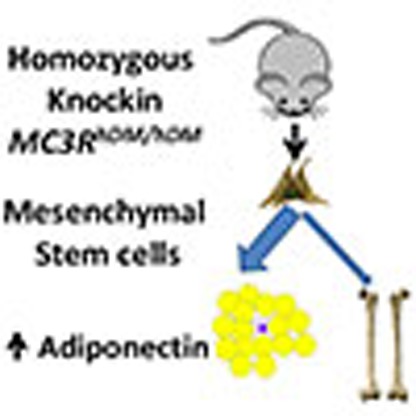

The murine melanocortin 3 receptor (*Mc3r*) plays an important role in regulating energy homeostasis^1^. Homozygous *Mc3r* knockout mice have a phenotype distinct from those of *ob/ob* or *Mc4r* knockouts^2^ because *Mc3r* knockout mice exhibit greater fat mass with reduced fat-free mass, so that total body weight is not notably increased. *Mc3r* knockout mice show greater feeding efficiency (the ratio of weight gain to energy intake); they are described as hypophagic or normophagic relative to controls and appear to maintain normal metabolic rate^1^. Recent studies have also reported impaired fasting-induced lipolysis and corticosterone secretion^3^ as well as potential dysregulation of mesolimbic dopaminergic reward systems^4^ in *Mc3r* knockout mice.

Linkage studies indicate that the human *MC3R* locus is associated with body weight^5^. Coding sequence variants that may be associated with obesity have also been reported in human *MC3R*^6^,^7^,^8^,^9^,^10^. A heterozygous missense sequence variant Ile183Asn (I183N) that inactivates *MC3R* function^11^,^12^ was identified in two individuals^7^. Other variants, including C17A (Thr6Lys) and G241A (Val81Ile) have not been found individually to affect signal transduction^12^ or be associated with body weight^6^,^8^,^9^,^10^. We and others have reported that homozygosity for the C17A+G241A *MC3R* haplotype is associated with childhood obesity and higher fat mass than observed for wild type or heterozygous children^13^,^14^,^15^, although this result has not been replicated among adults^16^ and some *in vitro* data suggest that the major start site for translation for *MC3R* may begin at a second in-frame ATG that is downstream of C17A, placing C17A in the 5′ untranslated region^17^,^18^. However, some *in vitro* studies have found translation is possible from the first ATG^19^ and have suggested that the C17A+G241A *hMC3R* may be partially inactive, with significantly fewer surface receptor binding sites, decreased signal transduction and less protein expression^13^,^15^. It remains unclear whether C17A+G241A *MC3R* affects energy homeostasis, given the genetic heterogeneity of humans in the extant studies. To characterize the potential effects of these gene variants on body weight, energy balance and metabolism, we therefore generated and compared two novel homozygous knock-in mouse models, replacing the murine *Mc3r* with either wild-type human *MC3R* (*MC3R*^*hWT/hWT*^) or the double-mutant C17A+G241A human *MC3R* (*MC3R*^*hDM/hDM*^), finding greater adiposity but without marked metabolic derangements in *MC3R*^*hDM/hDM*^.

## Results

### Validation of replacement of *Mc3r* in knock-in mice

**Figure 1:**
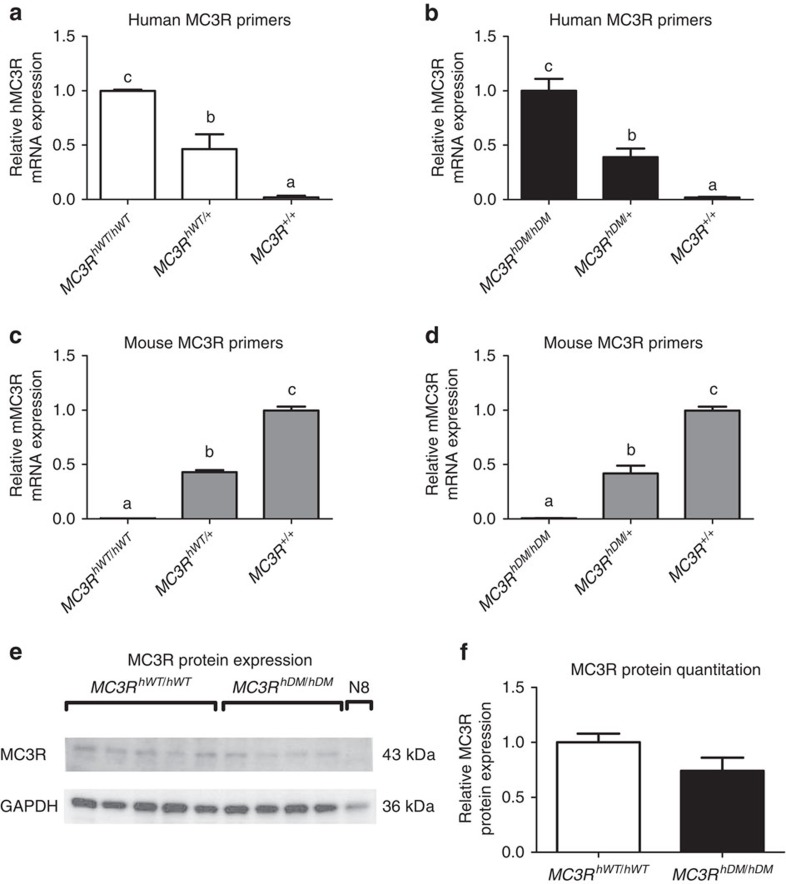
Validation of mouse *MC3R* replacement by human *MC3R*. Quantitative real-time PCR for relative hypothalamic mRNA expression normalized by β-actin expression by the 2–ΔΔCt method (*n*=3/group) in 4-month-old female C57BL/6 mice (*MC3R*^*+/+*^) or knock-in mice that were homozygous (*MC3R*^*hWT/hWT*^) or heterozygous (*MC3R*^*hWT/+*^) for the common alleles for the human *MC3R* or homozygous (*MC3R*^*hDM/hDM*^) or heterozygous (*MC3R*^*hDM/+*^) for the human *MC3R* sequence variants C17A+G241A. Expression was determined using human-specific *MC3R* primers (**a**,**b**) and mouse-specific *MC3R* primers (**c**,**d**). *MC3R* protein expression (**e**) was measured by western blotting (for *MC3R*^*hWT/hWT*^ *n*=5; for *MC3R*^*hDM/hDM*^ *n*=4) in homozygous mice and in the hypothalamic N8 murine cell line, which does not express MC3R mRNA. MC3R protein expression adjusted for GAPDH was quantified using Image J (**f**). Data are represented as mean±s.e.m. A different letter represents significant differences at *P*<0.05 compared with the other groups. Similar results were found for male mice (data not shown). Groups were compared by one-way analysis of variance followed by Bonferroni post-tests (**a**–**d**) and Student's *t*-test (two-tailed) (**f**).

The strategy and targeting vector used to replace murine *Mc3r* with human *MC3R* are shown in Supplementary Fig. 1. Direct sequencing after PCR amplification of genomic DNA of homozygous mice confirmed that murine *Mc3r* genes were replaced with human wild-type or double-mutant (C17A+G241A) *MC3R* and that there were no other genomic sequence differences introduced in the 6.17-kB targeted region. Only human *MC3R* messenger RNA (mRNA) expression in the hypothalamus was found for homozygous knock-in mice by quantitative RT–PCR performed using mouse- and human-specific primers (Fig. 1a–d). Western analysis confirmed MC3R was expressed in total hypothalamic protein lysates from both homozygous knock-in models (Fig. 1e,f).

### Body composition

**Figure 2:**
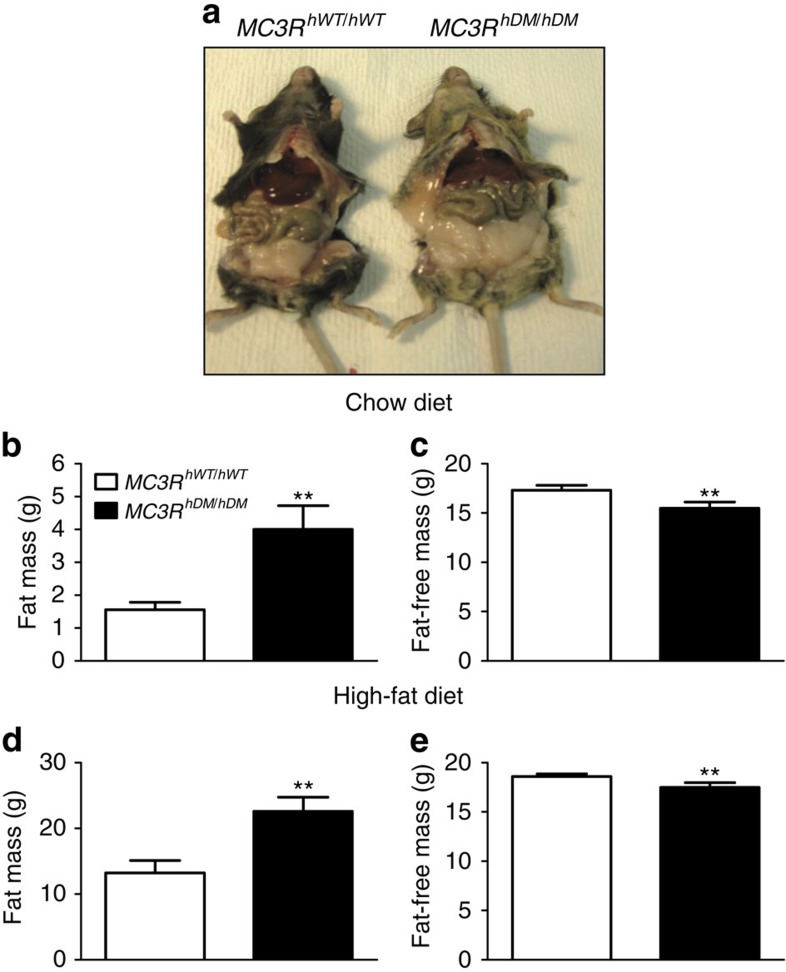
Greater fat mass and reduced fat-free mass in *MC3R*^*hDM/hDM*^ mice. (**a**) Female (5-month old) *MC3R*^*hDM/hDM*^ mice had increased adiposity compared with *MC3R*^*hWT/hWT*^. (**b**,**c**) Female *MC3R*^*hWT/hWT*^ (*n*=7, open bars) or *MC3R*^*hDM/hDM*^ (*n*=10, closed bars) mice were fed chow diet or (**d**,**e**) high-fat diet (*MC3R*^*hWT/hWT*^ *n*=6; *MC3R*^*hDM/hDM*^ *n*=9) for 2 months. Body fat mass and fat-free mass were measured by MRI at age 2–3 months and 4–5 months for chow-fed and high-fat-fed mice, respectively. Data are represented as mean±s.e.m. *P<0.05 and **P<0.01 *MC3R*^*hDM/hDM*^ versus *MC3R*^*hWT/hWT*^ mice. Groups were compared by Student's *t*-tests (two-tailed) (**b**–**e**).

To examine the effects of double-mutant human *MC3R* on body composition, we compared body weight and body composition of littermate female and male C57BL/6 mice without human gene insertion (*MC3R*^*+/+*^, *n*=16) with female and male knock-in mice that were heterozygous (*MC3R*^*hWT/+*^) or homozygous (*MC3R*^*hWT/hWT*^) for the wild-type human *MC3R* and with female and male mice that were heterozygous (*MC3R*^*hDM/+*^) or homozygous (*MC3R*^*hDM/hDM*^) for the double-mutant human *MC3R*. On chow diets, female and male *MC3R*^*hDM/hDM*^ mice had significantly greater body weight compared with *MC3R*^*hWT/hWT*^ or *MC3R*^*+/+*^ mice (Supplementary Fig. 2). No differences in body weight were observed among *MC3R*^*+/+*^, *MC3R*^*hWT/+*^, *MC3R*^*hWT/hWT*^ or *MC3R*^*hDM/+*^ mice (Supplementary Fig. 2). On a 45% high-fat diet, female and male *MC3R*^*hDM/hDM*^ mice also had significantly greater body weight compared with *MC3R*^*hWT/hWT*^ mice (Supplementary Fig. 3). Body composition was notably affected by *MC3R*^*hDM/hDM*^. On both chow and high-fat diets, both female (Fig. 2a–e) and male (Supplementary Fig. 4a–e) *MC3R*^*hDM/hDM*^ mice had significantly greater body fat mass, but reduced fat-free mass, compared with *MC3R*^*hWT/hWT*^ mice. The increased fat mass in high-fat-fed *MC3R*^*hDM/hDM*^ appeared not to be fat-depot specific, as evidenced by increased epididymal fat, subcutaneous flank fat and interscapular brown fat depots (Supplementary Fig. 5a–c). Although haematoxylin and eosin (H&E) staining showed that lipid accumulation appeared to be increased in brown adipose tissue of *MC3R*^*hDM/hDM*^, UCP1 protein expression was maintained in *MC3R*^*hDM/hDM*^ mice (Supplementary Fig. 6a–d). There was no significant difference in liver weight between high-fat-diet-treated mice (Supplementary Fig. 5d). H&E staining and liver triglyceride measurements after an overnight fast showed no significant differences in triglyceride accumulation between groups in the high-fat-fed condition (Supplementary Fig. 7a–c), suggesting that liver steatosis is not markedly exacerbated despite the greater fat mass of *MC3R*^*hDM/hDM*^ mice.

**Figure 3:**
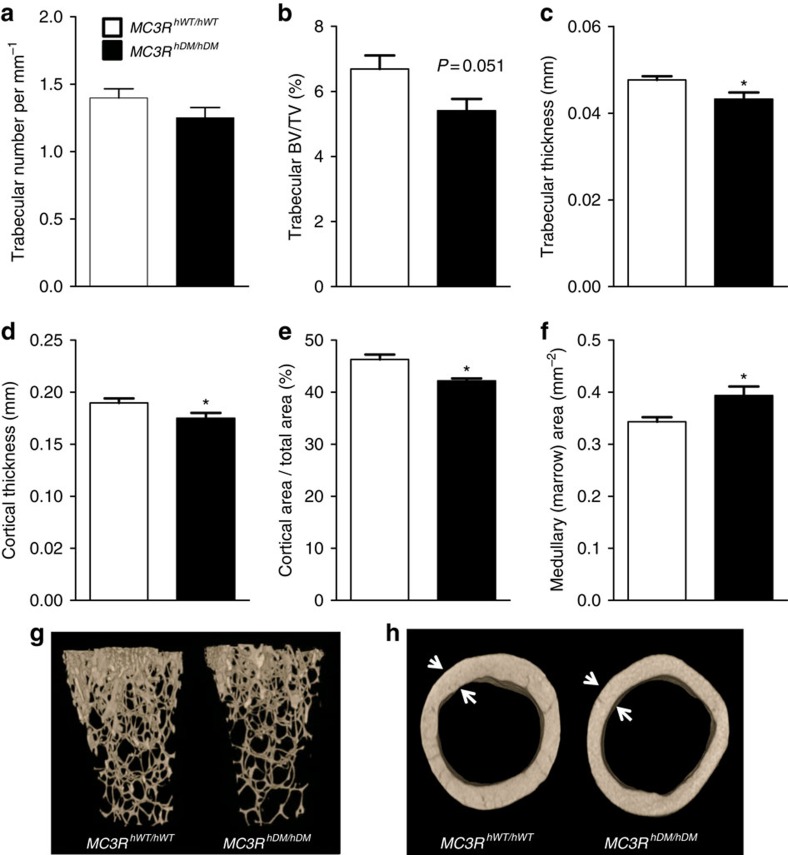
Micro-CT shows decreased bone in femurs of *MC3R*^*hDM/hDM*^ mice. (**a**) Trabecular number (per mm) of chow-fed female (12-week old) *MC3R*^*hDM/hDM*^ and *MC3R*^*hWT/hWT*^ mice. (**b**) Trabecular bone-volume fraction: Trabecular Bone Volume/Total Volume (BV/TV), (**c**) Trabecular thickness, (**d**) average cortical thickness, and (**e**) Cortical area per Total area fraction were reduced in *MC3R*^*hDM/hDM*^; (**f**) Medullary (marrow) area was increased in *MC3R*^*hDM/hDM*^; (**g**) representative reconstructed 3D images of femur trabecular bones; (**h**) representative cross-sectional cortical bones (**h**). Data are represented as mean±s.e.m.; *MC3R*^*hWT/hWT*^ *n*=7; *MC3R*^*hDM/hDM*^ *n*=8. *P<0.05 *MC3R*^*hDM/hDM*^ versus *MC3R*^*hWT/hWT*^ mice. Groups were compared by Student's *t*-tests (two-tailed) (**a**–**f**).

Crown-rump length of *MC3R*^*hDM/hDM*^ mice was slightly, but significantly, shorter than that of *MC3R*^*hWT/hWT*^ mice (Supplementary Fig. 8a,b). We used dual-energy X-ray absorptiometry (DXA) to further characterize body composition. Body fat mass was significantly increased without apparent differences in lean mass (Supplementary Fig. 9a–c), although percentage lean mass was significantly reduced in both female and male *MC3R*^*hDM/hDM*^ mice (Supplementary Fig. 9d). By DXA, we found no differences in total mouse bone mineral density (BMD; Supplementary Fig. 9e), although, in accord with their decreased length, total bone mineral content and bone area were significantly reduced in *MC3R*^*hDM/hDM*^ mice (Supplementary Fig. 9f,g). No skeletal malformations were identified in *MC3R*^*hDM/hDM*^ mice. To study bone microarchitecture more precisely, we performed micro computed tomography (CT) analysis using femurs from *MC3R*^*hWT/hWT*^and *MC3R*^*hDM/hDM*^ mice. Femurs from *MC3R*^*hDM/hDM*^ mice had reduced length (Supplementary Fig. 8c–e) as well as lower BMD in whole bone and trabecular bone without significant differences in cortical BMD (Supplementary Fig. 8f). Furthermore, *MC3R*^*hDM/hDM*^ mice exhibited a trend towards lower trabecular number and significantly decreased trabecular bone-volume fraction, trabecular bone thickness, cortical area fraction and average cortical thickness, compared with *MC3R*^*hWT/hWT*^ mice (Fig. 3a–e,g,h). Analysis of femurs (Fig. 3f) found the medullary cavity area in cortical regions was also significantly increased in *MC3R*^*hDM/hDM*^ mice, suggesting there might be increased marrow fat. We therefore isolated bone marrow from femurs, finding significantly greater marrow triglycerides in *MC3R*^*hDM/hDM*^ mice (Supplementary Fig. 10). These data indicate reduced bone mass and increased bone marrow fat in *MC3R*^*hDM/hDM*^compared to *MC3R*^*hWT/hWT*^ mice. Reduced bone size and increased marrow fat thus contribute to the decreased fat-free mass and increased fat mass of *MC3R*^*hDM/hDM*^ mice.

### Energy intake, feeding efficiency and energy expenditure

In both chow and high-fat diet-fed conditions, energy intake, assessed over 2 weeks and adjusted for fat-free mass, was significantly increased (*P*=0.044 and 0.048 for chow and high-fat diet-fed conditions, respectively) in female *MC3R*^*hDM/hDM*^ versus *MC3R*^*hWT/hWT*^ mice (Supplementary Table. 1a,b). Similar results were found in chow-fed male *MC3R*^*hDM/hDM*^ mice (data not shown). After adjusting for total body weight, energy intake was still significantly increased in high-fat-fed, but not chow-fed *MC3R*^*hDM/hDM*^ mice (Supplementary Table 1a,b). In both chow and high-fat diet-fed conditions, food intake was not different between groups after adjusting separately for both fat and fat-free mass (Supplementary Table 1a,b).

**Figure 4:**
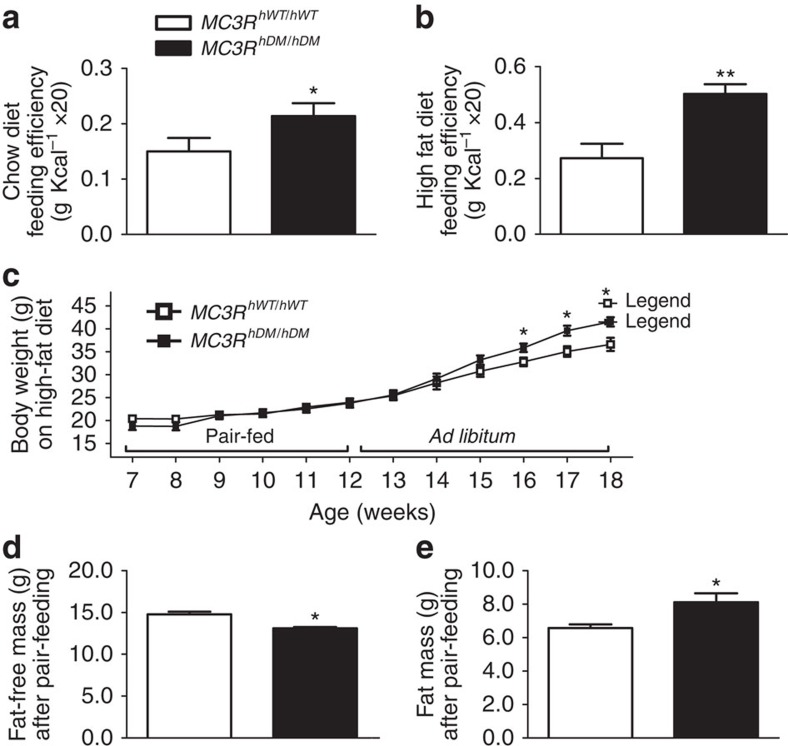
Increased feeding efficiency in *MC3R*^*hDM/hDM*^ mice. Feeding efficiency (the ratio of body weight to energy intake (g Kcal^−1^) × 20) was determined in female *MC3R*^*hWT/hWT*^ (open bars) and *MC3R*^*hDM/hDM*^ (closed bars) mice during (**a**) 5 weeks of chow diet feeding or (**b**) 7 weeks of high-fat-diet feeding. (**c**) For the high-fat pair-feeding study, the daily energy intake (11.83 Kcal per day) of 7- to 8-week-old female high-fat-fed *MC3R*^*hWT/hWT*^ mice (*n*=7) was supplied to the age matched female high-fat-fed *MC3R*^*hDM/hDM*^ mice (*n*=5), and their body weight change was monitored weekly. (**d**,**e**) Body composition of *MC3R*^*hWT/hWT*^ and *MC3R*^*hDM/hDM*^ mice was determined immediately after the end of pair-feeding period at age 12–13 weeks. Data are represented as mean±s.e.m. *P<0.05 and **P<0.01 *MC3R*^*hDM/hDM*^ versus *MC3R*^*hWT/hWT*^ mice. Groups were compared by Student's *t*-tests (two-tailed) (**a**,**b**,**d** and **e**) and two-way analysis of variance followed by Bonferroni post-tests (**c**).

Because increased feeding efficiency has been observed in *Mc3r* knockout mice^1^, we examined feeding efficiency in both female and male *MC3R*^*hWT/hWT*^ and *MC3R*^*hDM/hDM*^ mice fed *ad libitum*. Feeding efficiency (weight gain relative to energy intake) was markedly higher in female *MC3R*^*hDM/hDM*^ mice versus *MC3R*^*hWT/hWT*^ in both chow and high-fat-fed conditions (Fig. 4a,b). Similar results were found in male *MC3R*^*hDM/hDM*^ mice (data not shown). To examine feeding efficiency separately from energy intake, we pair-fed mice given a high-fat diet for 5 weeks. Body weight did not differ during pair feeding; however, fat mass was still increased, while fat-free mass was reduced in pair-fed *MC3R*^*hDM/hDM*^ mice versus *MC3R*^*hWT/hWT*^ (Fig. 4c–e). After switching from pair feeding to *ad libitum* diet, body weight significantly increased in *MC3R*^*hDM/hDM*^ mice compared with *MC3R*^*hWT/hWT*^ (Fig. 4c). These data indicate that alterations in both nutrient partitioning and energy intake contribute to the altered body composition and energy balance of *MC3R*^*hDM/hDM*^ mice.

To examine whether differences in energy expenditure contribute to the altered energy balance, we performed indirect calorimetry at 22 and 30 °C during chow and high-fat feeding using female *MC3R*^*hWT/hWT*^ *and MC3R*^*hDM/hDM*^ mice. During chow-feeding, total energy expenditure was significantly reduced in chow-fed *MC3R*^*hDM/hDM*^ mice at thermoneutrality after adjusting for body weight (Supplementary Table 2a). However, total energy expenditure was not significantly different among chow-fed mice after adjusting for fat-free mass or fat-free mass and fat mass at 22 or 30 °C (Supplementary Table 2a). During high-fat feeding (Supplementary Table 2b), *MC3R*^*hDM/hDM*^ mice, who exhibited no difference in total body weight compared with *MC3R*^*hWT/hWT*^, showed no significant change in total energy expenditure. Locomotor activity was also similar between groups during both chow and high-fat feeding (Supplementary Table 2a,b).

Respiratory exchange ratio (RER) was significantly lower at 22 °C in chow-fed *MC3R*^*hDM/hDM*^ mice (Supplementary Table 2a), but appeared to show a trend towards higher total RER (*P*=0.07) at 30 °C in high-fat-fed *MC3R*^*hDM/hDM*^ mice (Supplementary Table 2b), suggesting that altered substrate preference for generating energy does not appear to be a consistently applicable mechanism underlying the altered energy balance, since increased fat mass and reduced fat-free mass are found in both chow and high-fat-fed *MC3R*^*hDM/hDM*^ mice.

### Leptin action

**Figure 5:**
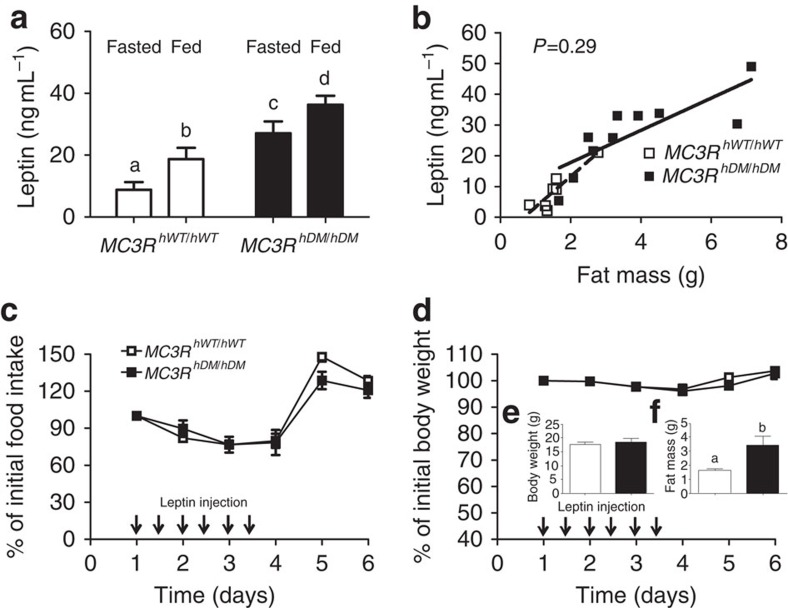
Maintained leptin sensitivity in *MC3R*^*hDM/hDM*^ mice. Blood was collected from tail veins of female *MC3R*^*hWT/hWT*^ (open symbols) and *MC3R*^*hDM/hDM*^ (closed symbols) mice. (**a**) Serum leptin concentrations were measured in fed (*n*=10 per group) and fasted (*n*=7 per group) conditions. (**b**) Serum leptin concentrations were significantly correlated with body fat mass in both *MC3R*^*hWT/hWT*^ (dotted line; *P*<0.0041) and *MC3R*^*hDM/hDM*^ (solid line, *P*<0.0043) mice, but these slopes were not significantly different (*P*=0.29) and leptin values were not different after results were adjusted for fat mass. (**c**,**d**) 2-month-old chow-fed mice (*n*=5 per group) were injected twice daily with mouse leptin (1 μg g^−1^ body weight, arrows) for 3 days. (**c**) Food intake and (**d**) body weight were measured daily. Inset bar graphs (**e**,**f**) show body weight and fat mass of the mice given leptin. Data are represented as mean±s.e.m. A different letter represents significant differences at *P*<0.05 compared with the other groups. Groups were compared by one-way (**a**) and two-way (**c**,**d**) analysis of variance followed by Bonferroni post-tests, Student's *t*-tests (two-tailed) (**e**–**f**), and analysis of covariance (**b**).

Leptin resistance is observed in mice with high-fat-diet-induced obesity and many other mouse obesity models. Serum leptin concentrations increase in obese states and are accompanied by decreased responsiveness to exogenous leptin administration^20^. Because the MC3R is believed to be a downstream target of leptin signalling^21^, we examined leptin responsiveness using female *MC3R*^*hWT/hWT*^ and *MC3R*^*hDM/hDM*^ mice. We found increased serum leptin concentrations under both fasting and fed conditions in *MC3R*^*hDM/hDM*^ mice (Fig. 5a). However, these differences were entirely accounted for by their increased fat mass (Fig. 5b). To examine whether decreased leptin responsiveness might explain the altered energy homeostasis of *MC3R*^*hDM/hDM*^ mice, we injected body-weight-matched *MC3R*^*hDM/hDM*^ and *MC3R*^*hWT/hWT*^ mice (7–8 weeks old) with leptin. At this age, *MC3R*^*hDM/hDM*^ mice already have greater fat mass than *MC3R*^*hWT/hWT*^ mice (Fig. 5e,f). The leptin-induced decreases in body weight and energy intake were not significantly different between groups (Fig. 5c,d), indicating maintained leptin sensitivity in *MC3R*^*hDM/hDM*^ despite their greater fat mass. These results suggest that inadequate leptin responsiveness does not explain why *MC3R*^*hDM/hDM*^ mice exhibit alterations in energy intake behaviour.

### Insulin sensitivity, serum lipid and hormone profiles

**Figure 6:**
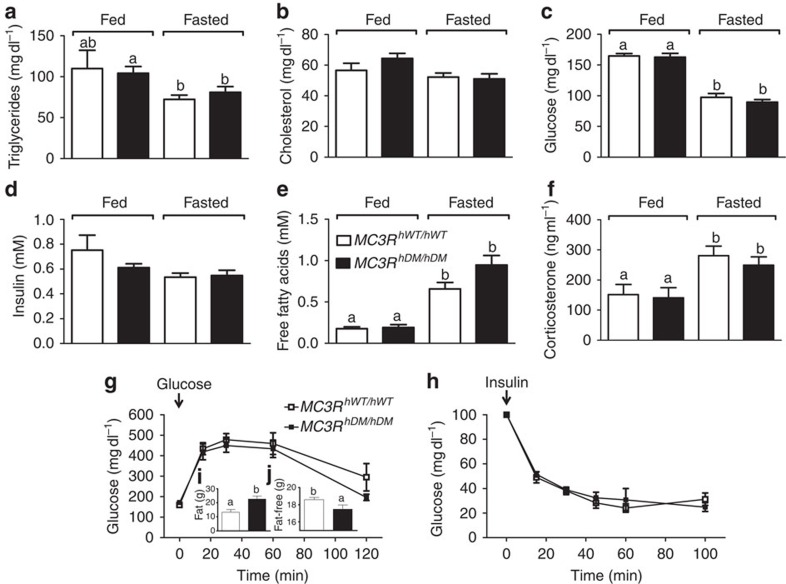
Maintained insulin sensitivity and circulating lipid and hormone profile in *MC3R*^*hDM/hDM*^ mice. Serum samples were used to measure (**a**) triglycerides, (**b**) total cholesterol, (**c**) glucose, (**d**) insulin, (**e**) free fatty acids and (**f**) corticosterone in 3-month-old female *MC3R*^*hWT/hWT*^ (open symbols) and *MC3R*^*hDM/hDM*^ (closed symbols) mice. For *MC3R*^*hWT/hWT*^ *n*=8 except *n*=6 for triglycerides and corticosterone; for *MC3R*^*hDM/hDM*^ *n*=9. For glucose (**g**) and insulin (**h**) challenge tests (*n*=7 per group), glucose and insulin were injected at time 0. Fat mass (**i**) and fat-free mass (**j**) were determined on the day of the glucose tolerance test. Data are represented as mean±s.e.m. A different letter represents significant differences at *P*<0.05 compared to the other groups. Groups were compared with one-way (**a**–**f**) and two-way (**g**–**h**) analysis of variance followed by Bonferroni post-tests and Student's **t**-tests (two-tailed) (**i**,**j**).

Obesity is generally accompanied by a dysmetabolic syndrome that involves alterations in the hormones and substrates associated with insulin resistance^22^,^23^. We examined whether obese *MC3R*^*hDM/hDM*^ mice have alterations in blood profile related to insulin sensitivity using female mice and found that serum triglycerides and cholesterol were, surprisingly, not significantly different in *MC3R*^*hWT/hWT*^ versus significantly more adipose *MC3R*^*hDM/hDM*^ mice examined under either chow-fed or fasted conditions (Fig. 6a,b). The serum concentrations of glucose and insulin were not significantly different between groups (Fig. 6c,d). Glucose and insulin-tolerance tests after high-fat diet feeding showed comparable responses for *MC3R*^*hDM/hDM*^ mice versus *MC3R*^*hWT/hWT*^ (Fig. 6g,h), suggesting that insulin sensitivity is relatively maintained in *MC3R*^*hDM/hDM*^ mice despite their greatly increased adiposity (Fig. 6i,j). Similarly, no significant differences in serum triglycerides, free fatty acids (FFA) and insulin-tolerance test results were found in male *MC3R*^*hDM/hDM*^ versus *MC3R*^*hWT/hWT*^ mice (data not shown).

It has been reported that a mechanism through which *MC3R* knockout mice increase fat mass and reduce lean mass is impaired fasting response, evidenced by lower fasting-induced adipose tissue lipolysis, higher basal corticosterone concentrations and diminished fasting-induced corticosterone^3^. However, basal FFA levels were not significantly different in the chow-fed condition, and fasting markedly increased serum FFA in both *MC3R*^*hWT/hWT*^ and *MC3R*^*hDM/hDM*^ mice (Fig. 6e). Similarly, fasting FFA concentrations were also not different between groups on a high-fat diet (Supplementary Fig. 11a). In addition, serum corticosterone was not different between groups in fasted and chow-fed conditions; fasting increased serum corticosterone in both *MC3R*^*hWT/hWT*^ and *MC3R*^*hDM/hDM*^ mice (Fig. 6f), indicating fasting-induced corticosterone response was maintained in *MC3R*^*hDM/hDM*^ mice.

### Adiponectin

**Figure 7:**
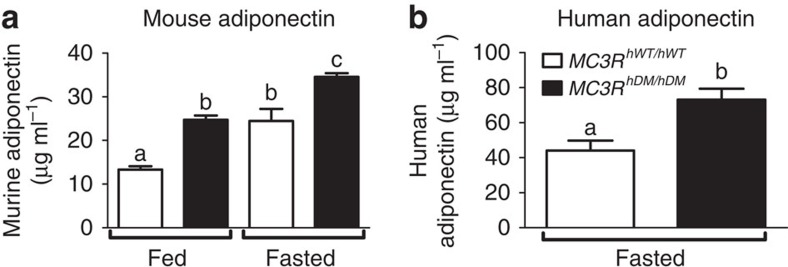
Increased serum adiponectin in *MC3R*^*hDM/hDM*^ mice and humans. (**a**) Serum adiponectin levels were measured in 3-month-old female *MC3R*^*hWT/hWT*^ (open bars) and *MC3R*^*hDM/hDM*^ (closed bars) mice under fed (*n*=8 per group) and fasted (*n*=11 per group). (**b**) Plasma samples from a matched cohort of *MC3R*^*hWT/hWT*^ and *MC3R*^*hDM/hDM*^ children (Supplementary Table 4) were analysed for adiponectin (*n*=13 per group). Data are represented as mean±s.e.m. A different letter represents significant differences at *P*<0.05 compared with the other groups. Groups were compared by one-way analysis of variance followed by Bonferroni post-tests (**a**) and analysis of covariance (**b**).

Adiponectin is an adipocyte-derived hormone whose gene expression and blood concentrations are usually inversely associated with adiposity and insulin resistance^24^,^25^,^26^. Unexpectedly, both female and male *MC3R*^*hDM/hDM*^ mice had significantly greater circulating adiponectin concentrations versus *MC3R*^*hWT/hWT*^ under both fasted and chow-fed conditions (Fig. 7a; Supplementary Fig. 11c) despite their notable increase in fat mass. On a high-fat diet, fat-mass-adjusted or non-adjusted fasting adiponectin was also significantly higher in female and male *MC3R*^*hDM/hDM*^ mice, respectively (Supplementary Fig. 11b,d). These data indicate that MC3R sequence variants (C17A+G241A) paradoxically increase circulating adiponectin regardless of sex or nutritional state.

These findings led us to measure serum adiponectin in a cohort (Supplementary Table 4) of age- and body-fat-matched children with homozygous wild type (*MC3R*^*hWT/hWT*^, *n*=13) or homozygous double-mutant (*MC3R*^*hDM/hDM*^, *n*=13) *MC3R*. Human subjects with *MC3R*^*hDM/hDM*^ also had significantly greater serum adiponectin than those with *MC3R*^*hWT/hWT*^ after adjusting for fat mass, sex and age (*P*<0.01, Fig. 7b).

### White adipose tissue adiponectin, PPARγ and phospho-AMPK

**Figure 8:**
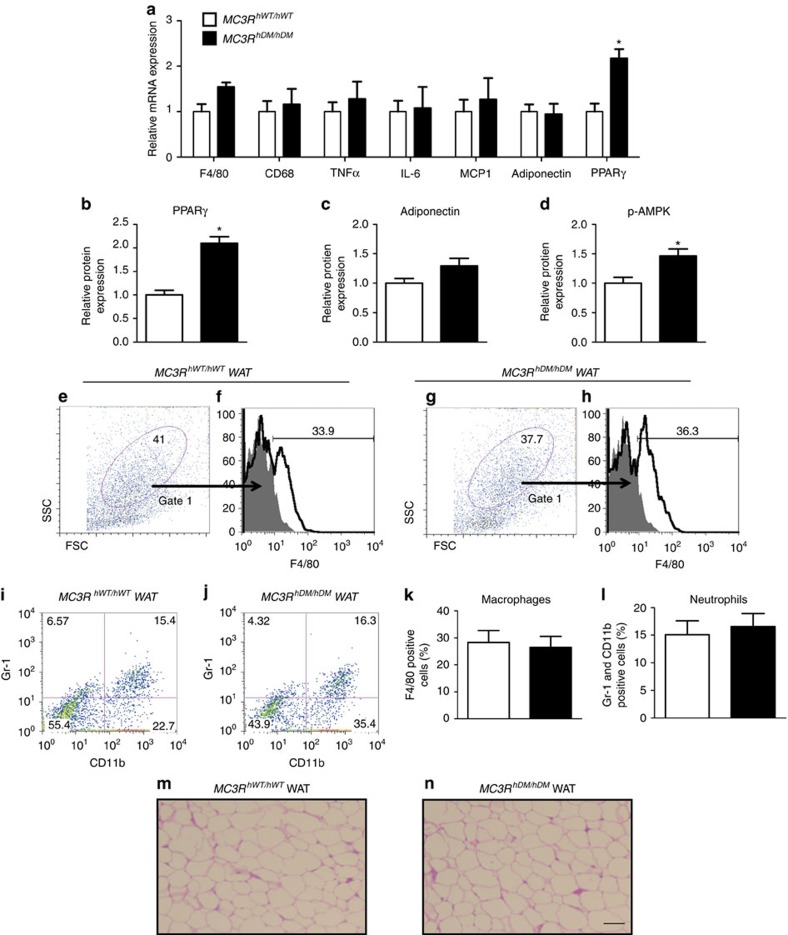
Maintained adipose tissue function in *MC3R*^*hDM/hDM*^ mice. Gonadal fat was isolated from fasted female mice fed a high-fat diet for (**a**) quantitative real-time PCR (*MC3R*^*hWT/hWT*^ *n*=5; *MC3R*^*hDM/hDM*^ *n*=8/group), (**b**–**d**) western blotting analysis (*MC3R*^*hWT/hWT*^ *n*=5; *MC3R*^*hDM/hDM*^ *n*=4), and (**e**–**l**) FACS analysis (*MC3R*^*hWT/hWT*^ *n*=4; *MC3R*^*hDM/hDM*^ *n*=5) in *MC3R*^*hWT/hWT*^ (open bars) and *MC3R*^*hDM/hDM*^ (closed bars) mice. (**a**) The expression levels of genes related to macrophage infiltration, inflammation and adipose tissue metabolism were normalized for β-actin expression. Western protein expression results for (**b**) peroxisome proliferator-activated receptor gamma (PPARγ), (**c**) adiponectin and (**d**) phosphospecific 5′-adenosine monophosphate-activated protein kinase (p-AMPK), divided by glyceraldehyde 3-phosphate dehydrogenase (GAPDH) expression and normalized relative to average for *MC3R*^*hWT/hWT*^. The stromal vascular fraction was isolated from gonadal fat (∼2 g) from *MC3R*^*hWT/hWT*^ and *MC3R*^*hDM/hDM*^mice. For detecting macrophages by flow cytometry, a gating strategy was used to enrich samples from *MC3R*^*hWT/hWT*^ and *MC3R*^*hDM/hDM*^mice for adipose tissue macrophages by selecting cells in the gate 1 area (**e**,**g**). The gate 1 area was stained with a F4/80 antibody (**f** and **h**) and the dot plots depict forward scatter (FSC) and side scatter (SSC) (left). For detecting neutrophils, whole cells (non-gated) were stained with CD11b and Gr-1 (**i**,**j**). Bar graphs show the average values±s.e.m. for percentage of cells in the stromal vascular fraction that were (**k**) macrophages and (**l**) neutrophils. *P<0.05 *MC3R*^*hDM/hDM*^versus *MC3R*^*hWT/hWT*^. (**m**,**n**) Haematoxylin and eosin stained sections showed no apparent differences in adipocyte morphology between groups. Groups were compared by Student's *t*-tests (two-tailed) (**a**–**d** and **k**–**i**). Scale bar, 100 μm. Data are represented as mean±s.e.m. for a-d, k, and l. FACS, fluorescence-activated cell sorting

Adiponectin is primarily secreted from white adipose tissues (WAT)^27^ and WAT adiponectin expression is usually reduced in obesity^26^. To investigate the mechanism underlying the paradoxically increased adiponectin in *MC3R*^*hDM/hDM*^ mice, we measured WAT adiponectin expression. WAT adiponectin mRNA (Fig. 8a) and protein (Fig. 8c) expression were not decreased in *MC3R*^*hDM/hDM*^ mice despite their greater fat mass. Maintained adiponectin expression and secretion from the markedly increased adipose tissue mass of *MC3R*^*hDM/hDM*^ mice may thus explain their increased circulating adiponectin.

The mRNA and protein levels of PPARγ, which is an essential factor for adipocyte differentiation, were significantly increased in WAT of *MC3R*^*hDM/hDM*^ mice (Fig. 8a,b). Furthermore, the protein level of phosphorylated AMPK, which induces fatty acid oxidation, was significantly increased in WAT of *MC3R*^*hDM/hDM*^ mice (Fig. 8d).

### White adipose tissue markers of inflammation

Macrophages^28^,^29^ and neutrophils^30^,^31^ infiltrate into WAT in obesity, and this infiltration is associated with the development of insulin resistance. To examine WAT inflammation, we performed fluorescence-activated cell sorting on stromal vascular fractions isolated from WAT as well as WAT quantitative gene expression analysis. Measures of macrophage- (F4/80-positive cells) and neutrophil- (Gr-1 and CD11b double-positive cells) infiltration were not different in WAT of high-fat-fed *MC3R*^*hWT/hWT*^and *MC3R*^*hDM/hDM*^ mice (Fig. 8e–l), despite the greater adiposity of *MC3R*^*hDM/hDM*^ mice. In addition, the expression levels of macrophage markers (F4/80 and CD 68) and inflammatory genes (tumour necrosis factor α, interleukin-6 and MCP1) were not significantly changed in WAT of *MC3R*^*hDM/hDM*^ versus *MC3R*^*hWT/hWT*^ mice (Fig. 8a). H&E staining also showed no apparent differences in adipocyte morphology between groups (Fig. 8m,n). After collagenase digestion and isolation, however, adipocytes from the WAT of *MC3R*^*hDM/hDM*^ had clearly increased lipid droplet size and cell diameter versus *MC3R*^*hWT/hWT*^ (182±20 versus 132±20 μM, *P*=0.0002).

### Differentiation of mesenchymal stem cells

**Figure 9:**
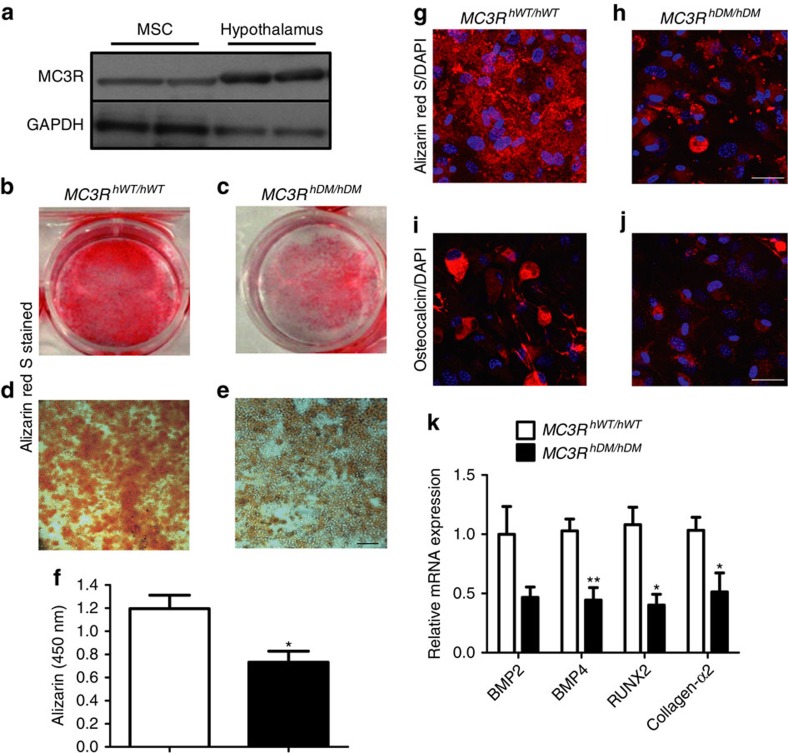
Decreased osteoblast differentiation in MSCs of *MC3R*^*hDM/hDM*^ mice. MSCs were isolated from compact bone of tibia and femur from chow-fed female *MC3R*^*hWT/hWT*^ and *MC3R*^*hDM/hDM*^ mice at 7 weeks of age. Cells were isolated from one mouse for each group, and six independent experiments were performed (*n*=6/group). Isolated MSCs were cultured in 25-cm cell culture dishes for 4 passages (see methods for detailed information). MSCs were used (**a**) for western blotting to examine MC3R protein expression or (**b**–**f**) MSCs were differentiated into osteoblasts for 14 days to examine differentiation capacity. (**b**–**c**) Cultured MSCs differentiated into osteoblasts after Alizarin red S staining. (**d**–**e**) Microscopic images (10X) of osteoblasts after Alizarin red S staining. Scale bar, 100 μm. (**f**) Stained Alizarin red S was extracted from osteoblast and quantified at 450 nm. (**g**–**j**) Confocal microscopic images of osteoblasts stained with Alizarin red S (red) (**g**,**h**) or osteocalcin (red) (**i**,**j**). Nuclei were stained with DAPI (blue). Representative maximum intensity projection images are shown. Scale bar, 25 μm. (**k**) qPCR analysis of genes related to osteoblast differentiation (7 days after differentiation). Similar results were found for male mice (data not shown). Data are represented as mean±s.e.m. for **f** and **k**. Groups were compared by Student's *t*-tests (two-tailed) (**f**,**k**).

**Figure 10:**
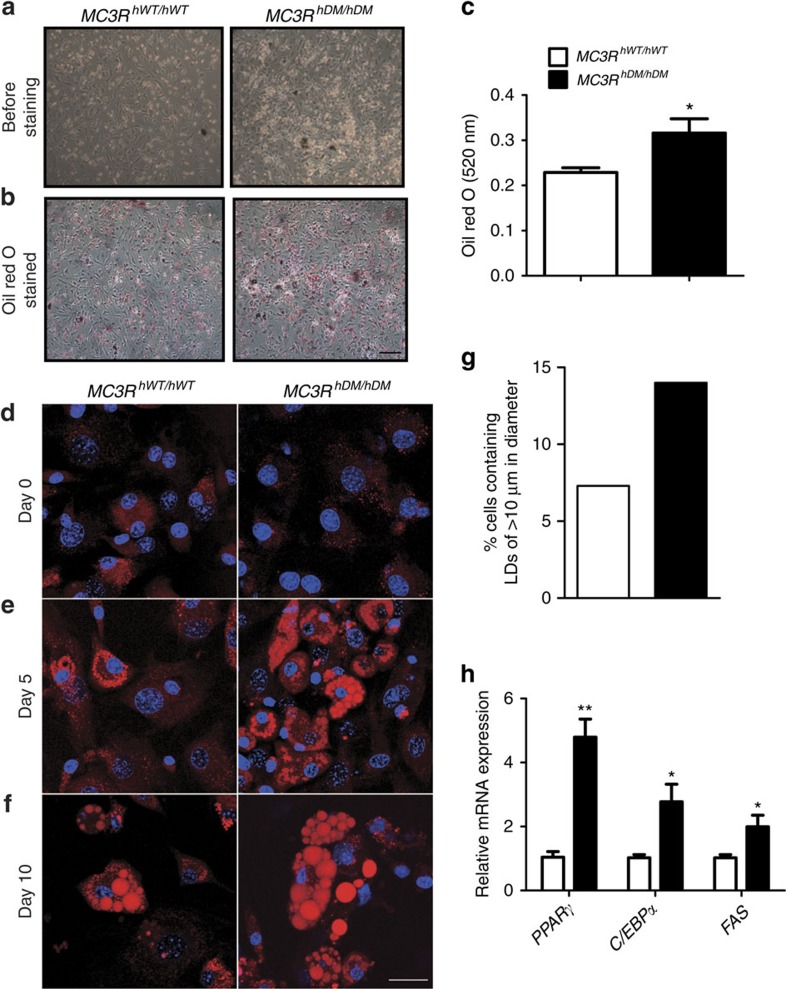
Increased adipocyte differentiation in MSCs of *MC3R*^*hDM/hDM*^ mice. MSCs were isolated as described in Fig. 8. (**a**–**f**) MSCs were differentiated into adipocytes to examine differentiation capacity. Cells were isolated from one mouse for each group, and six independent experiments were performed (*n*=6 per group). (**a**) Microscopic images (× 10) of MSCs differentiated into adipocytes for 18 days. (**b**) Microscopic images of differentiated adipocytes after Oil red O staining. Scale bar, 100 μm (**c**) Oil red O was extracted and quantified at 520 nm. (**d**–**f**) Confocal microscopic images of MSCs differentiated into adipocytes. Cells were stained with Nile Red and Hoechst 33342 at indicated days. Representative maximum intensity projection images of lipid droplets (LDs, red) and nuclei (blue) are shown. Scale bar, 25 μm (**g**) The percentage of cells displaying LDs>10 μm in diameter was quantified (*n*=∼350). (**h**) qPCR analysis of genes related to adipocyte differentiation (7 days after differentiation). Similar results were found for male mice (data not shown). Data are represented as mean±s.e.m. for **c** and **h**. Groups were compared by Student's *t*-tests (two-tailed) (**c**,**h**).

MSCs are multipotent progenitor cells that can be differentiated to lineages of mesenchymal tissues including bone, fat and muscle^32^. Because *MC3R*^*hDM/hDM*^ mice showed increased fat mass and decreased fat-free mass including a reduction in bone parameters including BMD and bone thickness, we hypothesized that *MC3R*^*hDM/hDM*^ mice have altered MSC differentiation capacity, with a bias towards adipocytic lipid storing cells, instead of osteoblastic bone-forming cells. We therefore studied MSC differentiation capacity in *MC3R*^*hDM/hDM*^ and *MC3R*^*hWT/hWT*^ mice. To avoid potential contamination with haematopoietic stem cells, we isolated MSCs from compact bone including tibiae and femora of mice and confirmed the purity of MSCs by flow cytometry (Supplementary Fig. 12). Western blotting showed that MC3R protein is expressed in MSCs (Fig. 9a). To investigate altered differentiation capacity, isolated MSCs were differentiated into osteoblasts or adipocytes and stained with Alizarin Red S (for calcium) and Oil Red O (for triglycerides). Differentiation into bone-forming osteoblasts was reduced (Fig. 9b–f) while differentiation into triglyceride-depositing adipocytes was significantly increased in MSCs of both male and female *MC3R*^*hDM/hDM*^ mice compared with their wild-type counterparts (Fig. 10a–c, data not shown for males). In addition, the mRNA expression of genes related to osteoblast differentiation such as BMP4, RUNX2 and collagen-α2 was significantly decreased (Fig. 9k) and the mRNA expression of genes related to adipocyte differentiation such as PPARγ, C/EBPα and FAS was markedly increased (Fig. 10h). Confocal imaging analysis further revealed that MSCs from *MC3R*^*hDM/hDM*^ mice produced significantly less extracellular calcium deposits compared with their wild-type counterparts during osteoblast differentiation, although the levels of intracellular osteocalcin (osteoblast marker) were not significantly different among the groups (Fig. 9g,j). In contrast, during adipocyte differentiation, markedly increased lipid droplet formation was observed in MSCs obtained from the compact bone of *MC3R*^*hDM/hDM*^ mice compared with MSCs from wild-type controls (Fig. 10d–f). Furthermore, the percentage of cells containing lipid droplets >10 μm in diameter was higher in cells derived from the MSCs of *MC3R*^*hDM/hDM*^ during adipocyte differentiation (Fig. 10g). To further confirm the increased adipogenic capacity of *MC3R*^*hDM/hDM*^, we studied triglyceride accumulation in cells derived from the stromal vascular fraction (SVF) of adipose tissue, which is a rich source of preadipocytes, MSCs, endothelial progenitor cells and immune cells^33^. To minimize effects from other cell populations, we used an MSC differentiation medium believed to specifically differentiate adipogenic precursor cells into adipocytes. Cells from *MC3R*^*hDM/hDM*^ SVF produced more lipid droplets after adipogenic stimulation compared to cells from *MC3R*^*hWT/hWT*^ mice (Supplementary Fig. 13a–e). This result is consistent with the increase in PPARγ expression we found in *MC3R*^*hDM/hDM*^ adipose tissue, since PPARγ is an essential factor for adipocyte differentiation from MSCs. These data further suggest there is increased adipogenic capacity in *MC3R*^*hDM/hDM*^ compared with *MC3R*^*hWT/hWT*^ mice.

## Discussion

To validate previous human studies that have suggested children with double-mutant (C17A+G241A) *MC3R* have greater fat mass^13^,^14^,^15^, we developed two humanized mouse models expressing human wild-type and double-mutant *MC3R*. We found that mice with *MC3R*^*hDM/hDM*^ had altered nutrient partitioning, with increased body fat in adipose tissue and the bone marrow, along with decreased crown-rump length and reduced fat-free mass that was at least partially due to an alteration of MSC fate towards lipid accumulation instead of bone formation. Compared with the WAT of *MC3R*^*hWT/hWT*^, the WAT of *MC3R*^*hDM/hDM*^ mice had increased protein expression of PPARγ and p-AMPK. Energy homeostasis was altered such that *MC3R*^*hDM/hDM*^ mice demonstrated somewhat greater energy intake but also greater feeding efficiency, as is observed in *Mc3r* knockout mice. Finally, despite their greater adiposity, and unlike most obese mouse models, *MC3R*^*hDM/hDM*^ mice had a reduction in expected obesity-associated metabolic dysfunction. *MC3R*^*hDM/hDM*^ mice had greater circulating adiponectin, well-maintained insulin sensitivity, liver triglycerides and blood levels of metabolites and hormones that were similar to those of considerably less adipose *MC3R*^*hWT/hWT*^ mice. In addition, the much larger WAT of *MC3R*^*hDM/hDM*^ mice did not show greater immune cell infiltration or inflammation versus the WAT of *MC3R*^*hWT/hWT*^ mice. These results suggest that the C17A+G241A human *MC3R* appears to stimulate an expansion of adipose tissue that is relatively metabolically healthy.

Energy homeostasis is determined by the balance between energy intake and expenditure. Only total energy expenditure at thermoneutrality (30 °C) was significantly reduced in chow-fed *MC3R*^*hDM/hDM*^ versus *MC3R*^*hWT/hWT*^ mice after adjusting for body weight, but the differences between groups were not significant after adjusting for fat-free mass or when animals were studied in the high-fat-fed condition, indicating that the contribution of lower energy expenditure to the altered energy homeostasis, if any, may be subtle. This result is also similar to our prior human study^14^ that found no differences in resting energy expenditure, total daily energy expenditure or RER among children with *MC3R*^*hWT/hWT*^ and *MC3R*^*hDM/hDM*^.

On the other hand, we found that energy intake was increased in *MC3R*^*hDM/hDM*^ mice during both chow and high-fat feeding. Furthermore, we found no differences in body weight versus *MC3R*^*hWT/hWT*^ mice during pair feeding, but an increase in body weight in *MC3R*^*hDM/hDM*^ mice after switching from pair feeding to *ad libitum* intake, indicating that energy intake contributes to the altered energy balance. These data are also concordant with our previous human findings, that energy intake at a buffet meal was significantly higher in children with *MC3R*^*hDM/hDM*^ compared with children who were heterozygous or homozygous for wild-type *MC3R*^14^. However, increased food intake is not the only mechanism underlying the altered energy metabolism in *MC3R*^*hDM/hDM*^ mice because body composition was already changed during pair feeding before there were differences in total body weight. These data suggested changes in peripheral energy partitioning might contribute to the altered energy metabolism of *MC3R*^*hDM/hDM*^ mice. In support of this hypothesis, it has been reported that increased feeding efficiency primarily contributes to the altered energy balance in *MC3R* knockout mice because energy intake is not consistently increased and energy expenditure is not altered in *MC3R* knockout mice^1^. In addition, when *Mc3r* is recovered only in the central nervous system of *Mc3r* knockout mice, such mice continue to demonstrate greater fat mass and less fat-free mass than controls^34^, indicating the potential importance of peripheral Mc3r signalling for altered nutrient partitioning. It is thus likely that changes in peripheral MC3R action contribute to the altered body composition of *MC3R*^*hDM/hDM*^ mice. We hypothesized that altered *Mc3r* signalling could direct pluripotent MSC differentiation away from lean tissues and towards adipose tissue formation. Indeed, osteogenesis was notably reduced and lipogenesis was increased in MSCs of *MC3R*^*hDM/hDM*^ mice. In addition, micro-CT analysis showed that femurs from *MC3R*^*hDM/hDM*^ mice were shorter and had significantly reduced trabecular BMD and trabecular/cortical bone area in comparison to *MC3R*^*hWT/hWT*^ mice. These data suggest that biasing MSC fate towards adipocytes instead of osteoblasts at least partially explains the increased fat mass and reduced bone mass of *MC3R*^*hDM/hDM*^ mice.

Multiple tissues may be used to isolate MSCs, including adipose tissue, bone and bone marrow. Our study showed that MSCs isolated from compact bone from *MC3R*^*hDM/hDM*^ mice differentiated more readily into adipocytes rather than osteoblasts, but it is still unclear whether MSCs from bone contribute to the increase in adipose tissue mass in *MC3R*^*hDM/hDM*^ mice. A human study indicates that bone marrow transplant can supply adipogenic progenitor cells to adipose tissue of the recipient^35^. Other studies indicate that MSCs can be mobilized and secreted into circulation^36^,^37^,^38^. These studies show the possibility that adipocyte precursor cells may be supplied through the circulation into adipose tissue. Furthermore, it has been reported that adiponectin stimulates MSC mobilization and secretion into circulation^39^. It is possible that increased adiponectin may stimulate MSC mobilization and provide new sources of adipocytes in adipose tissue of *MC3R*^*hDM/hDM*^ mice. Adipocyte differentiation was also increased in cells derived from the SVF of *MC3R*^*hDM/hDM*^ adipose tissue, showing the possibility that MSC characteristics in adipose tissue may also be changed in a manner similar to what we described for bone-derived MSCs.

The specific alterations in MC3R function induced by *MC3R*^*hDM/hDM*^ remain somewhat unclear. *In vitro* studies have suggested there is decreased MC3R protein expression and therefore partial inactivation of the MC3R after transient transfections of C17A+G241A *MC3R*^13^,^15^. However, other *in vitro* data suggest that C17A may actually reside in the 5′-UTR of the major *MC3R* transcript^17^,^18^. It is conceivable that C17A+G241A may change the balance of translation from the first and second in-frame ATGs and potentially interfere with MC3R function, for instance by altering its membrane localization^18^. Regardless, if the only change caused by *MC3R*^*hDM/hDM*^ were decreased, but not absent, MC3R signalling, one might expect a phenotype similar to heterozygous MC3R knockout mice, which are not described as having marked changes in body composition. *MC3R*^*hDM/hDM*^ does not appear to induce a functional knockout of MC3R; despite some phenotypic similarities, total *Mc3r* deficiency and *MC3R*^*hDM/hDM*^ regulate tissue metabolism uniquely. For example, unlike *Mc3r* knockout mice, *MC3R*^*hDM/hDM*^ mice, do not exhibit hyperglycaemia in low fat-fed conditions or increased inflammation in WAT in high-fat-fed conditions compared with wild-type mice^40^. Further studies are necessary to understand how the mutations studied may affect tissue-specific receptor expression, receptor stability, receptor trafficking and downstream signalling in both the central nervous system and peripheral tissues *in vivo*, but these results suggest a hitherto unrecognized role of MC3R signalling in adipocyte development.

Obesity is generally associated with an impaired metabolic profile that includes changes in the hormones and substrates associated with insulin resistance^22^,^23^. However, *MC3R*^*hDM/hDM*^ mice maintained serum lipid concentrations and insulin sensitivity comparable to those of *MC3R*^*hWT/hWT*^ mice despite their increased fat mass and reduced fat-free mass. Our previous human study reported that the much more obese subjects we studied with *MC3R*^*hDM/hDM*^ had serum glucose, triglycerides, total cholesterol and low-density lipoprotein cholesterol levels that were not significantly different from those of considerably less obese *MC3R*^*hWT/hWT*^ children, although leptin and insulin were increased in proportion to fat mass among those with *MC3R*^*hDM/hDM*^^13^. The mechanisms through which obese *MC3R*^*hDM/hDM*^ humans and mice avoid obesity-related metabolic dysfunction remain to be further established. The MSC-fate-driven expansion of metabolically healthy adipose tissue due to greater differentiation of stem cells into young adipocytes may contribute to this phenomenon, because it has been shown that the recruitment of adipogenic stem cells provides healthy adipose tissue expansion that is not involved in inflammation and systemic insulin resistance^41^.

Adiponectin is an adipocyte-derived hormone that can improve insulin sensitivity and decrease inflammation^42^,^43^, whose gene expression and blood concentrations are normally inversely associated with body fat^25^. Surprisingly, despite the greater fat mass of *MC3R*^*hDM/hDM*^ mice, we found increased serum adiponectin. We confirmed the same finding in a cohort of humans with *MC3R*^*hDM/hDM*^ and *MC3R*^*hWT/hWT*^ who were matched for demographic and anthropometric variables including body mass index and adiposity. It has been proposed that high adiponectin may serve as a ‘starvation' signal in the hypothalamus that increases energy intake and reduces energy expenditure, thereby, counteracting leptin-mediated inhibition of energy intake^44^. Consistent with this view, a prior study has suggested that energy intake is a main regulator of circulating adiponectin^45^. The present study found that *MC3R*^*hDM/hDM*^ mice maintained consistently higher adiponectin concentrations than *MC3R*^*hWT/hWT*^ in both fasted and fed conditions. We also found that *MC3R*^*hDM/hDM*^ mice exhibited higher leptin in circulation, but it was related to their enlarged adipose tissue rather than to the presence of primary leptin resistance. Thus, proximal leptin signalling pathway defects do not appear to contribute to the altered energy intake behaviour of *MC3R*^*hDM/hDM*^ mice. It remains unclear why *MC3R*^*hDM/hDM*^ mice have increased energy intake. One intriguing possibility is that the increased adiponectin of *MC3R*^*hDM/hDM*^ mice may be mechanistically related to their greater food consumption and may play a role in maintaining their metabolic profile. Consistent with this hypothesis, when adiponectin is overexpressed in leptin-deficient mice, improvements in metabolic profile are accompanied by a remarkable expansion of adipose tissue^46^. *Ob/ob* mice that modestly overexpress adiponectin have increased PPARγ mRNA expression in WAT along with notable increases in WAT mass compared to *ob/ob* mice without adiponectin overexpression. The *ob/ob* mice with greater adiponectin also have ameliorated glucose and lipid profiles and reduced liver triglyceride and adipose tissue inflammation compared with *ob/ob* mice without adiponectin overexpression^46^. Similarly, we found that *MC3R*^*hDM/hDM*^ mice showed increased PPARγ mRNA and protein expression in WAT, as well as WAT immune cell infiltration and inflammatory cytokine expression that were not exaggerated compared with that of less adipose *MC3R*^*hWT/hWT*^ mice.

The role of MC3R in regulating inflammation is not fully understood. However, some studies suggest that inhibiting MC3R signalling may potentially be beneficial to reduce inflammation. A clinical study found that during resistance training, which has been shown to reduce chronic inflammation, decreased MC3R expression in monocytes was significantly correlated with reduced C-reactive proteins levels, independent of changes in adiposity^47^. In addition, delayed macrophage infiltration is observed in WAT of high-fat-fed *Mc3r* knockout mice compared with *Mc4r* knockout mice^40^,^48^. Whether MC3R directly regulates immune cell function needs to be further elucidated.

In conclusion, knock-in mice homozygous for human *MC3R* C17A+G241A have notably increased fat mass and reduced fat-free mass including decreased bone formation due to both greater energy intake and altered energy partitioning caused by MSC differentiation that is biased towards lipid-accumulating cells. These data help explain prior findings in humans of greater adiposity and energy intake among those homozygous for *MC3R* C17A+G241A. In mice, and at least in some respects among humans, the obese phenotype of *MC3R*^*hDM/hDM*^ is associated with an amelioration of the metabolic dysfunction usually associated with obesity. Our study confirms the importance of MC3R signalling in human energy homeostasis and metabolism and suggests a unique mechanism underlying the early increase in fat mass observed in *MC3R*^*hDM/hDM*^ mice and children with these *MC3R* mutations.

## Methods

### Generation of human *MC3R* knock-in mice

Mouse *Mc3r* was replaced by human wild-type or human double-mutant (C17A+G241A) *MC3R* (Supplementary Fig. 1). The constructs for generating the two knock-in mouse lines were prepared by recombineering^49^. In brief, a targeting vector was constructed in the vector PKO Scrambler 916 (Stratagene, La Jolla CA). This vector contains a 5′ diphtheria toxin A negative selection cassette as well as a 3′ neomycin positive selection cassette that is flanked by loxP sites. At the ‘homology 1' site (just 3′ to the negative selection cassette), we placed 2.978-kb genomic DNA (gDNA), comprising 1.895 kb of 5′ murine SV129 gDNA sequence upstream of the murine *Mc3r* start codon and 1.083 kb of the human *MC3R* sequence (either wild-type or C17A+G241A *MC3R)*. At the ‘homology 2' site (3′ to the positive selection cassette), we placed 3.192 kb of 3′ murine SV129 genomic DNA sequence starting immediately after the mouse *Mc3r* stop codon. C57BL/6 ES cells were electroporated with the targeting vector and selected using G418. Positive clones were injected into C57BL/6 blastocysts to produce chimeras. Chimeric male mice were then mated with C57BL/6 female mice, and germline transmission was assessed by PCR. Mice with germline transmission of the knock-in alleles were then mated with C57BL/6- Gt*(ROSA)26Sor*tm16(Cre)Arte mice (Taconic, Hudson, NY) to delete the neomycin cassette, followed by further crossing with >10 generations of C57BL/6 nontransgenic (*MC3R*^*+/+*^) mice, thereby creating heterozygous human *MC3R* double-mutant and wild-type mice in the C57BL/6 background. Heterozygous mice were crossed to obtain humanized homozygous, heterozygous and no construct mice. Genome scans (The Jackson laboratories, Bar Harbor, ME) with 155 single-nucleotide polymorphisms confirmed >99.5% identity with C57BL/6 for both knock-in mouse lines. All mice were maintained on a 12-h light, 12-h dark cycle and studied at 21–25 °C unless noted otherwise. Mice were fed either a chow diet (4.7% fat as calories, diet 7017, Harlan Laboratories, Frederick MD) or 45% high-fat diet (diet D12451, Research Diets, New Brunswick, NJ). All animal studies were conducted in accord with accepted standards of humane animal care under protocols approved by the NICHD or NIDDK Animal Care and Use Committees. There were no randomized or blinded experiments conducted.

### Identification of human plasma samples for adiponectin determination

A cohort of 13 *MC3R*^*hDM/hDM*^ and 13 *MC3R*^*hWT/hWT*^ children was identified from among participants of prior clinical research studies (http://www.clinicaltrials.gov/ct/show/NCT00001522 and http://www.clinicaltrials.gov/ct/show/NCT00001723) for whom a fasting plasma sample and body composition analysis by DXA were available for analysis. The human groups were selected to have similar age, race, sex, body mass index and adiposity (Supplementary Table 4). All children gave written assent and parents gave written consent. The studies were approved by the Institutional Review Board of the Eunice Kennedy Shriver National Institute of Child Health and Human Development.

### Mouse body composition, indirect calorimetry and locomotor activity

Body composition was determined using an EchoMRI mouse scanner (EchoMRI, Houston, TX) or using a PIXImus dual-energy X-ray absorptiometer (Lunar, Madison, WI), as indicated. Energy expenditure was measured at 22 and 30 *°*C using a four-chamber Oxymax system (Columbus Instruments, Columbus, OH) as previously described, with one mouse per chamber^50^. On day 1 to day 2, mice were acclimated in the metabolic chamber at room temperature. On day 3, metabolic parameters were measured at 22 *°*C for 24 h. On day 4, the temperature in the chamber was raised to 30 *°*C in the morning, and measurements were begun 1 h later. Locomotor activity was determined at the same time that energy expenditure was measured using infrared beam interruption^50^.

### Micro-CT scanning

To determine bone microarchitecture, the femurs from 12-week-old female *MC3R*^*hWT/hWT*^ and *MC3R*^*hDM/hDM*^ mice were isolated and scanned. Micro-CT was performed with the assistance of the NIH Mouse Imaging Facility using a Bruker MicroCT SkyScan 1172 Micro X-ray CT scanner (Micro Photonics, Inc. Allentown PA, USA, and Bruker MicroCT, Kontich, Belgium) with the X-ray source (focal spot size, 4 μm, energy range 20–100 kV) biased at 44 kV/267 μA and with a 0.5 mm Aluminium filter to reduce sample induced beam hardening. The images were acquired with a pixel size of 4.97 μm. Projections were acquired with an angular resolution of 0.5° through (180° or 360°) degrees rotation. Eight frames were averaged for each projection radiograph with an exposure time of 2,600 ms per frame. The scan duration was ∼2 h. Tomographic images were reconstructed filtered back projection using vendor-supplied software based on the Feldkamp cone beam algorithm. The data were calculated from the region of interest, which was defined as 0.503 mm offset from the growth plate; 2.753 mm height for trabecular bone, and 5.485 mm offset from growth plate; 0.418 mm height for cortical bone. The trabecular bone data include BMD, bone-volume fraction (BV/TV), trabecular number, trabecular thickness and trabecular separation. For cortical bone, data include total bone area (Tt.Ar), cortical area (Ct.Ar) and cortical area fraction (Ct.Ar/Tt.Ar) and mean cortical thickness.

### Immunoblots and quantitative real-time PCR

For immunoblotting, mechanically homogenized tissue samples were separated using 4–12% NuPAGE gels (Invitrogen, Carlsbad, CA). Protein was blotted with antibodies for MC3R (SC 8990, Santa Cruz Biotechnology, Dallas, TX), PPARγ (SC 7273, Santa Cruz), GAPDH (SC 25778, Santa Cruz), p-AMPK (2535, Cell Signaling Technology, Danvers, MA) and adiponectin (MAB 1119, R&D Systems, Minneapolis, MN). Primary antibodies were diluted to 1:1000 concentration. Protein levels were semiquantified by image density scanning using Image J analysis (NIH, Bethesda, MD). The values were adjusted for GAPDH expression. For measuring MC3R protein expression, the hypothalamic N8 murine cell line, which does not express MC3R was used as a negative control^51^.

Total RNA was prepared from gonadal fat and hypothalamus and homogenized with Trizol (Invitrogen, Carlsbad, CA). Genomic DNA was removed using RNAqueous kit (Ambion, Austin, TX) and complementary DNA was synthesized using SuperScript III first-strand (Invitrogen). Quantitative real-time PCR was performed using a 7900HT fast real-time PCR system (Applied Biosystems, Foster City, CA). For *MC3R, BMP2, BMP4, RUNX2* and *collagen-α2* gene expression, the Taqman gene expression system was used (HS 00252036 for Human *MC3R*, Mm 00434876 for mouse *Mc3r*, Mm 01340178 for *BMP2*, Mm 00432087 for *BMP4*, Mm 00501584 for *RUNX2* and Mm 00483888 for *collagen-α2*, Life Technologies, Grand Island, NY). For other gene expression studies, SYBR qPCR (4367659, Life Technologies, Grand Island, NY) was performed using the primers listed in Supplementary Table 3.

### Flow cytometry

Mouse gonadal fat pads (∼2 g) were minced and digested for 50 min at 37 °C with collagenase D (1 mg ml^−1^; Roche Applied Science, Indianapolis, IN) in DMEM containing 10% fetal bovine serum (pH 7.4) and 1% bovine serum albumin. After filtration of the digested fat through a nylon mesh (100 μM), the filtrate was centrifuged at 1,000 r.p.m. for 5 min. The SVF was obtained from the resulting pellet. SVF counts were determined with the Guava Viacount reagent in a Guava EasyCyte Mini System (Millipore, Billerica, MA). A gating strategy was used to enrich samples from *MC3R*^*hWT/hWT*^ and *MC3R*^*hDM/hDM*^ mice for adipose tissue macrophages by selecting cells in the gate 1 area (see Fig. 8e,g)^52^. The proportion of macrophages was detected using mouse F4/80 antibody conjugated with fluorescein isothiocyanate (eBioscience, San Diego, CA) and the proportion of neutrophils was detected using mouse Gr-1 conjugated with fluorescein isothiocyanate, and mouse CD11b conjugated with phycoerythrin (eBioscience). Flow cytometry was conducted with a Guava EasyCyte Mini System (Millipore) using Guava CytoSoft Version 4.2.1 (Millipore) and FlowJo, Version 7 (TreeStar, Ashland, OR).

### Analysis of circulating metabolites and hormones

Blood was collected from mouse tail veins cut with a scalpel blade in the fed or overnight-fasted (from 1800 hours to 0800 hours) condition and serum samples were prepared by centrifugation (4 °C) for 1,000–2,000*g* for 10 min. Glucose was measured using a Glucometer Elite (Bayer, Elkhart, IN). Other metabolites or hormones were measured using the indicated kits; Insulin (SRI-13K, Linco Research, St Charles, MO), corticosterone (ADI-900-097; Enzo Life science, Farmingdale, NY), IGF-1 (22-IGF-R21, ALPCO, Salem, NH), Adiponectin (DRP 300 for human plasma samples obtained by venipuncture and MRP300 for mouse serum, R&D SYSTEMS, Minneapolis, MN), Leptin (MOB00, R&D SYSTEMS), triglycerides (337-B; Sigma), and non-esterified fatty acids (13831175; Roche Molecular Biochemicals, Indianapolis, IN).

### Triglyceride measurement

Small pieces of liver were taken (∼0.3 g) for triglyceride measurement. For determination of bone marrow triglyceride content, both intact femurs were collected. The tips of the bones were cut to allow insertion of a 20-G needle at one end. Using a syringe, 20 ml of PBS was injected to flush bone marrow cells out of the marrow cavity and into a 50-ml conical tube. Cells were centrifuged at 1,200 r.p.m. for 5 min at room temperature. Eighten volumes of Hexane/2-propanol (3:2) solvent was added to each sample (liver and flushed bone marrow) followed by homogenization with tissue grinder. The lipid-containing layer was transferred to a new tube. After evaporation, the dried extract was reconstituted with 2-propanol. Triglycerides were measured using L-type TG M Microtiter Procedure (Wako Diagnostics, Richmond, VA, reagents 461-08992, 461-09092, 464-01601).

### Pair feeding, leptin treatment and glucose and insulin-tolerance tests

A 5-week high-fat pair-feeding study was performed by supplying the daily energy intake (11.83 Kcal per day) of 7- to 8-week-old female high-fat-fed *MC3R*^*hWT/hWT*^ mice to 7- to 8-week-old female high-fat-fed *MC3R*^*hDM/hDM*^ mice. To study leptin sensitivity, mice were injected twice daily with mouse leptin (R&D Systems, Minneapolis, MN, 1 μg g^−1^ body weight, at 0730 hours and 1800 hours) for 3 days. To examine glucose homeostasis, glucose (2 g kg^−1^) or insulin (0.75 U kg^−1^) was injected intraperitoneally into fasted (from 1800 hours to 0800 hours for glucose) or fed (for insulin) female mice. Glucose was measured with a glucometer (Glucometer Elite, Bayer, Elkhart, IN).

### Mesenchymal stem cell isolation from mouse compact bone

Mouse compact bones including tibiae and femurs were used to isolate MSCs rather than bone marrow tissues because hematopoietic cells still exist in bone-marrow-derived MSC cultures even after the preparation has been passaged nine times^53^. To obtain MSCs, 7- to 8-week-old mice were killed and soaked with 100 ml of 70% (vol/vol) ethanol for 5 min. Tibiae and femurs were isolated from both legs. The muscles and tendons were removed from tibiae and femurs using microdissecting scissors. Bone marrow was flushed out of the bones and the bones were minced with heavy-duty scissors (18054-11, Fine Science Tools, Foster City, CA) and digested with collagenase D (Roche Diagnostics, Indianapolis, IN). The digested bone fragments were cultured in 25-cm cell culture flasks with MSC basal media (Stem Cell Technologies, Vancouver, Canada) supplemented with MSC stimulatory supplement (Stem Cell Technologies, Vancouver, Canada) until it was passaged four times. Passaged MSCs underwent evaluation of purity by flow cytometry using CD29 (12-0291-83, ebioscience, San Diego, CA), CD106 (561613, ebioscience), CD44 (15-0441-83, ebioscience) and CD105 (12-1051-81, ebioscience) to detect pure MSCs and CD34 (primitive haematopoietic progenitor and endothelial cell marker) and CD45 (pan-leukocyte marker) for negative MSC markers (11-0341-85 and 15-0451-81, ebioscience) (Supplementary Fig. 12). MSCs were differentiated into osteoblasts with osteogenic media (CCM 009, R&D system, Minneapolis, MN) for 14 days and into adipocytes with adipogenic media for 18 days (CCM 011, R&D system, Minneapolis, MN). Differentiated osteoblasts and adipocytes were stained with Alizarin Red S and Oil Red O respectively. Alizarin Red S and Oil Red O were then extracted from the cells using an osteogenesis quantitation kit (ECM 815, Millipore, Billerica, MA) and isopropanol respectively and quantified at 450 nm for Alizarin Red S and 520 nm for Oil Red O.

### Confocal microscopy

Isolated MSCs (6 × 10^4^cells per well) were plated in Nunc Lab-Tek 1.5 Chambered 4 well Coverglass (Thermo Fisher Scientific Inc., Waltham, MA) and differentiated into adipocytes or osteoblasts as described earlier. At differentiation day 0, day 5 and day 10, cells were incubated with 0.00001% (vol/vol) Nile Red (Life Technologies, Grand Island, NY) and 1 μg ml^−1^ Hoechst 33342 (Life Technologies, Grand Island, NY) at 37 °C for 20 min to stain lipid droplets and nuclei, respectively. Afterwards, the cells were rapidly washed three times with prewarmed culture media. For osteoblast imaging, cells were fixed by using 4% paraformaldehyde in PBS at 37 °C for 15 min. After washing three times in PBS, cells were stained with Alizarin Red S as described earlier, or were blocked with PBS containing 3% bovine serum albumin and 0.3% TritonX100 (PBS-T) for 1 h at room temperature. Then, cells were incubated with 1/50 diluted osteocalcin antibody (SC 30045, Santa Cruz Biotechnology, Dallas, TX) in PBS-T at 4 °C for overnight. After washing three times in PBS for 5 min, the cells were treated with 1/500 diluted Alexa Fluor-conjugated secondary antibody (A 11060, Life Technologies) at 37 °C for 1 h. Then, the cells were incubated with 1 μg ml^−1^ DAPI (Life Technologies, Grand Island, NY) in PBS and were rinsed three times in PBS for 5 min. Images were acquired using Leica TCS SP5 (Leica Microsystems Inc., Buffalo Grove, IL). For imaging Nile Red and Hoechst 33342 (or DAPI), 561 nm white-light laser and 405-nm diode laser were used for excitation, and the emission signals were collected between 635–700 and 480–500 nm, respectively. For imaging Alizarin Red and endogenous osteocalcin, 546 nm white-light laser were used for excitation, and the emission signals were collected between 580 and 700 nm. ImageJ^54^ was used to process and analyse images.

### Primary preadipocyte culture and adipocyte differentiation

Epididymal adipose tissue was collected from 4- to 5-month-old female mice. Tissue was minced and incubated in DMEM with type I collagenase (30 mg per 4 g of tissue) at 37 °C for 1 h with gentle shaking. Samples were diluted 1:1 ratio into 10% FBS/DMEM after digestion, followed by filtration with 100 and 40 μM cell strainers to remove undigested tissues. Cells were centrifuged at 600 *g* for 7 min and the upper lipid layer and supernatant were discarded. Pellets were resuspended in 10 ml of 10% FBS/DMEM media, and 3–4 × 10^5^ cells were plated into collagen pre-coated six-well plates. Media was changed on the next day and every 3 days until cells were differentiated into adipocytes. After 2 weeks, a special adipogenic medium (CCM 011, R&D system, Minneapolis, MN) was added for 2 weeks until Oil Red O staining.

### Statistical analysis

Sample sizes were based on prior animal studies suggesting meaningful results from 5–15 animals per group. Data are expressed as mean±s.e.m. unless otherwise indicated. GraphPad Prism 5.0 software (GraphPad Software, San Diego, CA) was used for Student's *t*-tests (two-tailed) and two-way analysis of variance followed by Bonferroni post-tests. IBM SPSS 18 software (Armonk, New York) was used for performing analysis of covariance for human adiponectin adjusted for age, sex and percentage fat mass and for adjusted energy intake and expenditure models for mice. Differences were considered significant at *P*<0.05. Data met assumptions of the statistical tests including requirements for similar variance across groups. Data are represented as mean±s.e.m. unless otherwise indicated.

## Additional information

**How to cite this article:** Lee, B. *et al*. A mouse model for a partially inactive obesity-associated human *MC3R* variant. *Nat. Commun*. 7:10522 doi: 10.1038/ncomms10522 (2016).

## Supplementary Material

Supplementary Information Supplementary Figures 1-13 and Supplementary Tables 1-4

## References

[b1] Chen A. S. . Inactivation of the mouse melanocortin-3 receptor results in increased fat mass and reduced lean body mass. Nat. Genet. 26, 97–102 (2000). 10973258 10.1038/79254

[b2] Butler A. A. The melanocortin system and energy balance. Peptides 27, 281–290 (2006). 16434123 10.1016/j.peptides.2005.02.029 PMC2735083

[b3] Renquist B. J. . Melanocortin-3 receptor regulates the normal fasting response. Proc. Natl Acad. Sci. USA 109, E1489–E1498 (2012). 22573815 10.1073/pnas.1201994109 PMC3384161

[b4] Lippert R. N., Ellacott K. L. & Cone R. D. Gender-specific roles for the Melanocortin 3 receptor in the regulation of the mesolimbic dopamine system in mice. Endocrinology 155, 1718–1727 (2014). 24605830 10.1210/en.2013-2049 PMC3990839

[b5] Lee J. H. . Genome scan for human obesity and linkage to markers in 20q13. Am. J. Hum. Genet. 64, 196–209 (1999). 9915959 10.1086/302195 PMC1377718

[b6] Hani E. H. . Naturally occurring mutations in the melanocortin receptor 3 gene are not associated with type 2 diabetes mellitus in French Caucasians. J. Clin. Endocrinol. Metab. 86, 2895–2898 (2001). 11397906 10.1210/jcem.86.6.7589

[b7] Lee Y. S., Poh L. K. & Loke K. Y. A novel melanocortin 3 receptor gene (MC3R) mutation associated with severe obesity. J. Clin. Endocrinol. Metab. 87, 1423–1426 (2002). 11889220 10.1210/jcem.87.3.8461

[b8] Li W. D. . Melanocortin 3 receptor (MC3R) gene variants in extremely obese women. Int. J. Obes. Relat. Metab. Disord. 24, 206–210 (2000). 10702772 10.1038/sj.ijo.0801114

[b9] Schalin-Jantti C. . Melanocortin-3-receptor gene variants in morbid obesity. Int. J. Obes. Relat. Metab. Disord. 27, 70–74 (2003). 12532156 10.1038/sj.ijo.0802184

[b10] Wong J. . Melanocortin-3 receptor gene variants in a Maori kindred with obesity and early onset type 2 diabetes. Diabetes. Res. Clin. Pract. 58, 61–71 (2002). 12161058 10.1016/s0168-8227(02)00126-2

[b11] Rached M., Buronfosse A., Begeot M. & Penhoat A. Inactivation and intracellular retention of the human I183N mutated melanocortin 3 receptor associated with obesity. Biochim. Biophys. Acta 1689, 229–234 (2004). 15276649 10.1016/j.bbadis.2004.03.009

[b12] Tao Y. X. & Segaloff D. L. Functional characterization of melanocortin-3 receptor variants identify a loss-of-function mutation involving an amino acid critical for G protein-coupled receptor activation. J. Clin. Endocrinol. Metab. 89, 3936–3942 (2004). 15292330 10.1210/jc.2004-0367

[b13] Feng N. . Co-occurrence of two partially inactivating polymorphisms of MC3R is associated with pediatric-onset obesity. Diabetes 54, 2663–2667 (2005). 16123355 10.2337/diabetes.54.9.2663 PMC1861848

[b14] Savastano D. M. . Energy intake and energy expenditure among children with polymorphisms of the melanocortin-3 receptor. Am. J. Clin. Nutr. 90, 912–920 (2009). 19656839 10.3945/ajcn.2009.27537 PMC2744620

[b15] Lee Y. S., Poh L. K., Kek B. L. & Loke K. Y. The role of melanocortin 3 receptor gene in childhood obesity. Diabetes 56, 2622–2630 (2007). 17639020 10.2337/db07-0225

[b16] Matsuoka N. . Association of MC3R with body mass index in African Americans. Int. J. Body Comp. Res. 5, 123–129 (2007).

[b17] Tarnow P., Rediger A., Schulz A., Gruters A. & Biebermann H. Identification of the translation start site of the human melanocortin 3 receptor. Obes. Facts 5, 45–51 (2012). 22433616 10.1159/000336070

[b18] Park J., Sharma N. & Cutting G. R. Melanocortin 3 receptor has a 5' exon that directs translation of apically localized protein from the second in-frame ATG. Mol. Endocrinol. 28, 1547–1557 (2014). 25051171 10.1210/me.2014-1105 PMC4154237

[b19] Schioth H. B., Muceniece R., Wikberg J. E. & Szardenings M. Alternative translation initiation codon for the human melanocortin MC3 receptor does not affect the ligand binding. Eur. J. Pharmacol. 314, 381–384 (1996). 8957262 10.1016/s0014-2999(96)00566-3

[b20] Myers M. G. Jr . Challenges and opportunities of defining clinical leptin resistance. Cell Metab. 15, 150–156 (2012). 22326217 10.1016/j.cmet.2012.01.002 PMC3281561

[b21] Shimizu H., Inoue K. & Mori M. The leptin-dependent and -independent melanocortin signaling system: regulation of feeding and energy expenditure. J. Endocrinol. 193, 1–9 (2007). 17400797 10.1677/JOE-06-0144

[b22] Boden G. Obesity, insulin resistance and free fatty acids. Curr. Opin. Endocrinol., Diabetes Obes. 18, 139–143 (2011). 21297467 10.1097/MED.0b013e3283444b09 PMC3169796

[b23] Clement K. & Vignes S. [Inflammation, adipokines and obesity]. Rev. Med. Interne. 30, 824–832 (2009). 19394723 10.1016/j.revmed.2009.03.363

[b24] Lee B. & Shao J. Adiponectin and energy homeostasis. Rev. Endocr. Metab. Disord. 15, 149–156 (2013). 24170312 10.1007/s11154-013-9283-3 PMC4006341

[b25] Lihn A. S., Pedersen S. B. & Richelsen B. Adiponectin: action, regulation and association to insulin sensitivity. Obes. Rev. 6, 13–21 (2005). 15655035 10.1111/j.1467-789X.2005.00159.x

[b26] Kern P. A., Di Gregorio G. B., Lu T., Rassouli N. & Ranganathan G. Adiponectin expression from human adipose tissue: relation to obesity, insulin resistance, and tumor necrosis factor-α expression. Diabetes 52, 1779–1785 (2003). 12829646 10.2337/diabetes.52.7.1779

[b27] Ahima R. S. Adipose tissue as an endocrine organ. Obesity 14, (Suppl 5), 242S–249S (2006). 17021375 10.1038/oby.2006.317

[b28] Patsouris D. . Ablation of CD11c-positive cells normalizes insulin sensitivity in obese insulin resistant animals. Cell. Metab. 8, 301–309 (2008). 18840360 10.1016/j.cmet.2008.08.015 PMC2630775

[b29] Weisberg S. P. . Obesity is associated with macrophage accumulation in adipose tissue. J. Clin. Invest. 112, 1796–1808 (2003). 14679176 10.1172/JCI19246 PMC296995

[b30] Elgazar-Carmon V., Rudich A., Hadad N. & Levy R. Neutrophils transiently infiltrate intra-abdominal fat early in the course of high-fat feeding. J. Lipid. Res. 49, 1894–1903 (2008). 18503031 10.1194/jlr.M800132-JLR200

[b31] Talukdar S. . Neutrophils mediate insulin resistance in mice fed a high-fat diet through secreted elastase. Nat. Med. 18, 1407–1412 (2012). 22863787 10.1038/nm.2885 PMC3491143

[b32] Pittenger M. F. . Multilineage potential of adult human mesenchymal stem cells. Science 284, 143–147 (1999). 10102814 10.1126/science.284.5411.143

[b33] Riordan N. H. . Non-expanded adipose stromal vascular fraction cell therapy for multiple sclerosis. J. Transl. Med. 7, 29 (2009). 19393041 10.1186/1479-5876-7-29 PMC2679713

[b34] Begriche K. . Genetic dissection of the functions of the Melanocortin-3 receptor, a seven-transmembrane g-protein-coupled receptor, suggests roles for central and peripheral receptors in energy homeostasis. J. Biol. Chem. 286, 40771–40781 (2011). 21984834 10.1074/jbc.M111.278374 PMC3220494

[b35] Ryden M. . Transplanted bone marrow-derived cells contribute to human adipogenesis. Cell. Metab. 22, 408–417 (2015). 26190649 10.1016/j.cmet.2015.06.011

[b36] Dalle Carbonare L., Valenti M. T., Zanatta M., Donatelli L. & Lo Cascio V. Circulating mesenchymal stem cells with abnormal osteogenic differentiation in patients with osteoporosis. Arthritis. Rheum. 60, 3356–3365 (2009). 19877060 10.1002/art.24884

[b37] Marketou M. E. . Circulating mesenchymal stem cells in patients with hypertrophic cardiomyopathy. Cardiovasc. Pathol. 24, 149–153 (2015). 25744383 10.1016/j.carpath.2015.02.005

[b38] Roufosse C. A., Direkze N. C., Otto W. R. & Wright N. A. Circulating mesenchymal stem cells. Int. J. Biochem. Cell Biol. 36, 585–597 (2004). 15010325 10.1016/j.biocel.2003.10.007

[b39] Yu L. . Adiponectin regulates bone marrow mesenchymal stem cell niche through a unique signal transduction pathway: an approach for treating bone disease in diabetes. Stem Cells 33, 240–252 (2015). 25187480 10.1002/stem.1844 PMC4681406

[b40] Trevaskis J. L. . Role of adiponectin and inflammation in insulin resistance of Mc3r and Mc4r knockout mice. Obesity 15, 2664–2672 (2007). 18070757 10.1038/oby.2007.318 PMC2753182

[b41] Sun K., Kusminski C. M. & Scherer P. E. Adipose tissue remodeling and obesity. J. Clin. Invest. 121, 2094–2101 (2011). 21633177 10.1172/JCI45887 PMC3104761

[b42] Berg A. H., Combs T. P. & Scherer P. E. ACRP30/adiponectin: an adipokine regulating glucose and lipid metabolism. Trends Endocrinol. Metab. 13, 84–89 (2002). 11854024 10.1016/s1043-2760(01)00524-0

[b43] Fantuzzi G., Mazzone T. & Matsuzawa Y. in Adipose Tissue and Adipokines in Health and Disease Humana Press (2007).

[b44] Kubota N. . Adiponectin stimulates AMP-activated protein kinase in the hypothalamus and increases food intake. Cell. Metab. 6, 55–68 (2007). 17618856 10.1016/j.cmet.2007.06.003

[b45] Qiao L., Lee B., Kinney B., Yoo H. S. & Shao J. Energy intake and adiponectin gene expression. Am. J. Physiol. Endocrinol. Metab. 300, E809–E816. 21325106 10.1152/ajpendo.00004.2011 PMC3093972

[b46] Kim J. Y. . Obesity-associated improvements in metabolic profile through expansion of adipose tissue. J. Clin. Invest. 117, 2621–2637 (2007). 17717599 10.1172/JCI31021 PMC1950456

[b47] Henagan T. M., Forney L., Dietrich M. A., Harrell B. R. & Stewart L. K. Melanocortin receptor expression is associated with reduced CRP in response to resistance training. J. Appl. Physiol. 113, 393–400 (2012). 22678961 10.1152/japplphysiol.00107.2012 PMC4422369

[b48] Ellacott K. L., Murphy J. G., Marks D. L. & Cone R. D. Obesity-induced inflammation in white adipose tissue is attenuated by loss of melanocortin-3 receptor signaling. Endocrinology 148, 6186–6194 (2007). 17901224 10.1210/en.2007-0699

[b49] Copeland N. G., Jenkins N. A. & Court D. L. Recombineering: a powerful new tool for mouse functional genomics. Nat. Rev. Genet. 2, 769–779 (2001). 11584293 10.1038/35093556

[b50] Jiang C. . Disruption of hypoxia-inducible factor 1 in adipocytes improves insulin sensitivity and decreases adiposity in high-fat diet-fed mice. Diabetes 60, 2484–2495 (2011). 21873554 10.2337/db11-0174 PMC3178277

[b51] Belsham D. D. . Generation of a phenotypic array of hypothalamic neuronal cell models to study complex neuroendocrine disorders. Endocrinology 145, 393–400 (2004). 14551229 10.1210/en.2003-0946

[b52] Vandanmagsar B. . The NLRP3 inflammasome instigates obesity-induced inflammation and insulin resistance. Nat. Med. 17, 179–188. 21217695 10.1038/nm.2279 PMC3076025

[b53] Zhu H. . A protocol for isolation and culture of mesenchymal stem cells from mouse compact bone. Nat. Protoc. 5, 550–560 (2010). 20203670 10.1038/nprot.2009.238

[b54] Schneider C. A., Rasband W. S. & Eliceiri K. W. NIH Image to ImageJ: 25 years of image analysis. Nat. Methods. 9, 671–675 (2012). 22930834 10.1038/nmeth.2089 PMC5554542

