# Supplementary material for: A mouse model for a partially inactive obesity-associated human MC3R variant
Source: Nat Commun. 2016 Jan 28;7:10522. doi: 10.1038/ncomms10522 (PMC4738366; doi:10.1038/ncomms10522)
Supplement: Supplementary Information — Supplementary Figures 1-13 and Supplementary Tables 1-4 [file ncomms10522-s1.pdf]

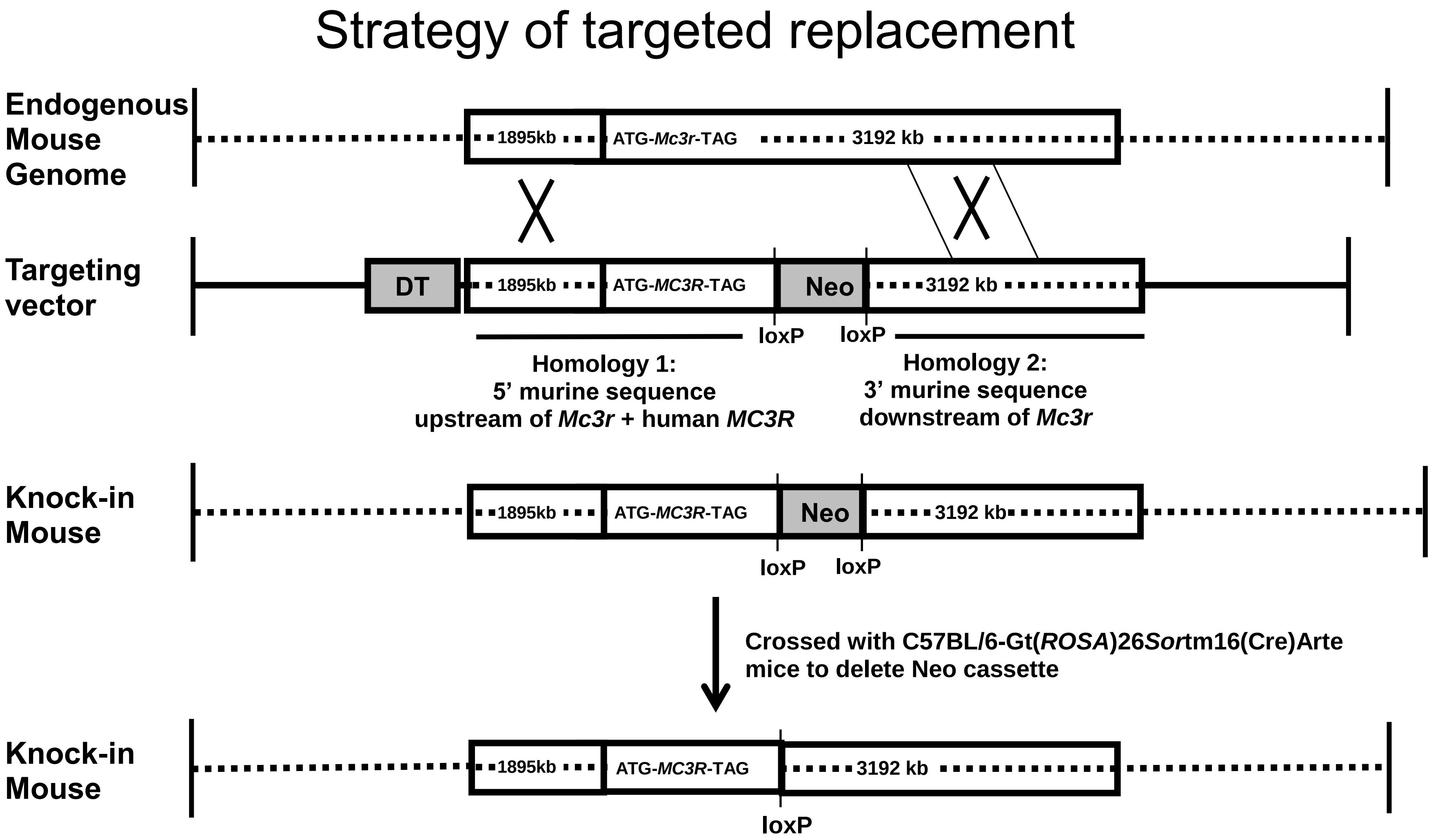

Supplementary Figure 1. Strategy of targeted replacement for generating human *MC3R* knock-in mouse models. DT: diphtheria toxin negative selection cassette; Neo: neomycin positive selection cassette. Dotted lines represent endogenous murine genomic sequence, solid lines represent targeting vector sequence. *Mc3r* - Mouse *Mc3r*. *MC3R* - Human *MC3R.* Note: size not to scale.

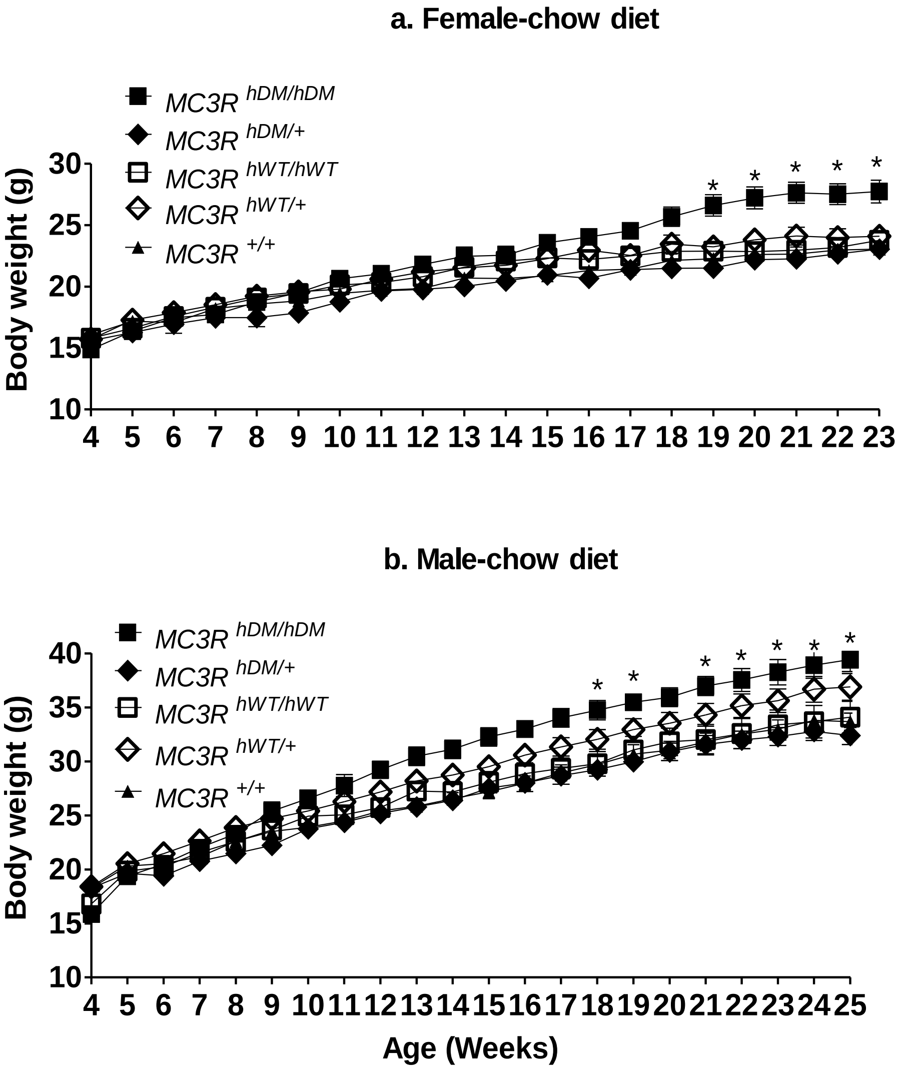

Supplementary Figure 2. Increased body weight found only in *MC3R*$^{hDM/hDM}$ mice. Body weight was measured weekly in (a) female and (b) male *MC3R*$^{+/+}$ (n=16), *MC3R*$^{hWT/hWT}$ (n=16), *MC3R*$^{hWT/+}$ (n=16), *MC3R*$^{hDM/+}$ (n=16), and *MC3R*$^{hDM/hDM}$ (n=11) mice in chow-fed condition. Significant differences compared to *MC3R*$^{+/+}$ were found only for *MC3R*$^{hDM/hDM}$, but not for other genotypes. Data are represented as mean ± SEM. 2-way ANOVA followed by Bonferroni post-tests were performed. *$P<0.05$ *MC3R*$^{hDM/hDM}$ vs. MC3R$^{hWT/hWT}$ or *MC3R*$^{+/+}$.

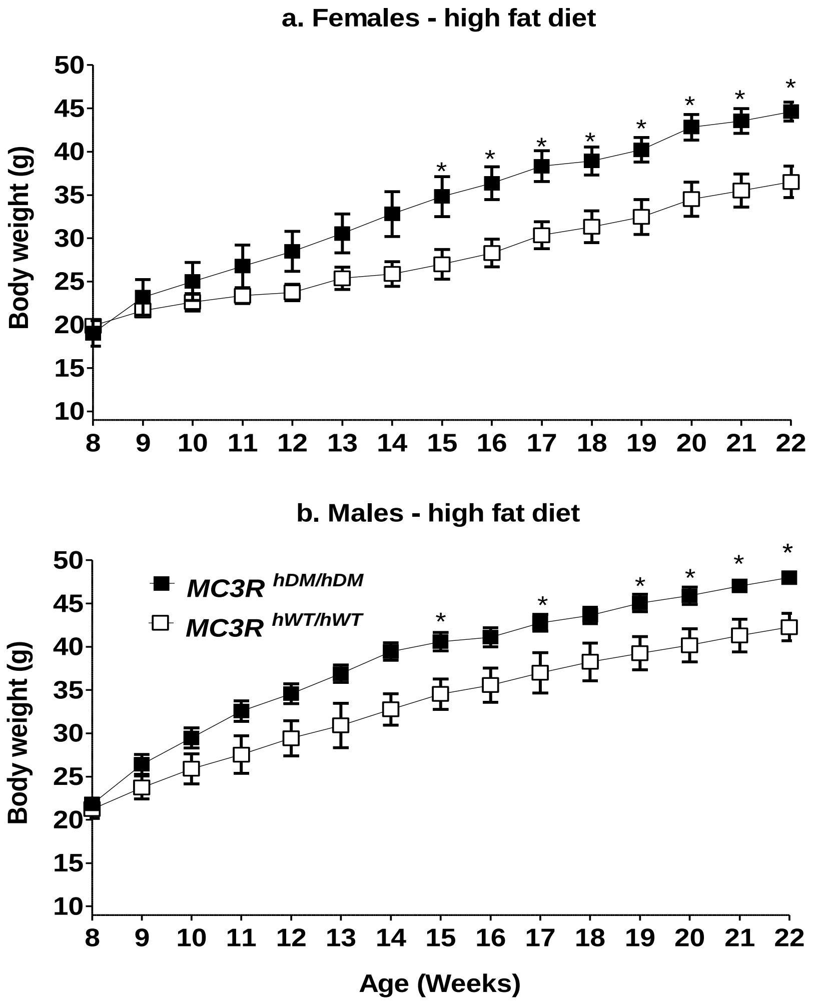

Supplementary Figure 3. Increased body weight in high-fat-fed *MC3R*$^{hDM/hDM}$ mice.
(a) Female and (b) male *MC3R*$^{hWT/hWT}$, and *MC3R*$^{hDM/hDM}$ mice were fed a 45% high-fat diet for 15 weeks (for females, *MC3R*$^{hWT/hWT}$ n=11; *MC3R*$^{hDM/hDM}$ n=6; for males, *MC3R*$^{hWT/hWT}$ n=9 *MC3R*$^{hDM/hDM}$ n=11). Body weight was measured weekly. Data are represented as mean ± SEM. *$P<0.05$ *MC3R*$^{hDM/hDM}$ vs. *MC3R*$^{hWT/hWT}$ mice. 2-way ANOVA followed by Bonferroni post-tests were performed. *$P<0.05$ *MC3R*$^{hDM/hDM}$ vs. MC3R$^{hWT/hWT}$ or *MC3R*$^{+/+}$.

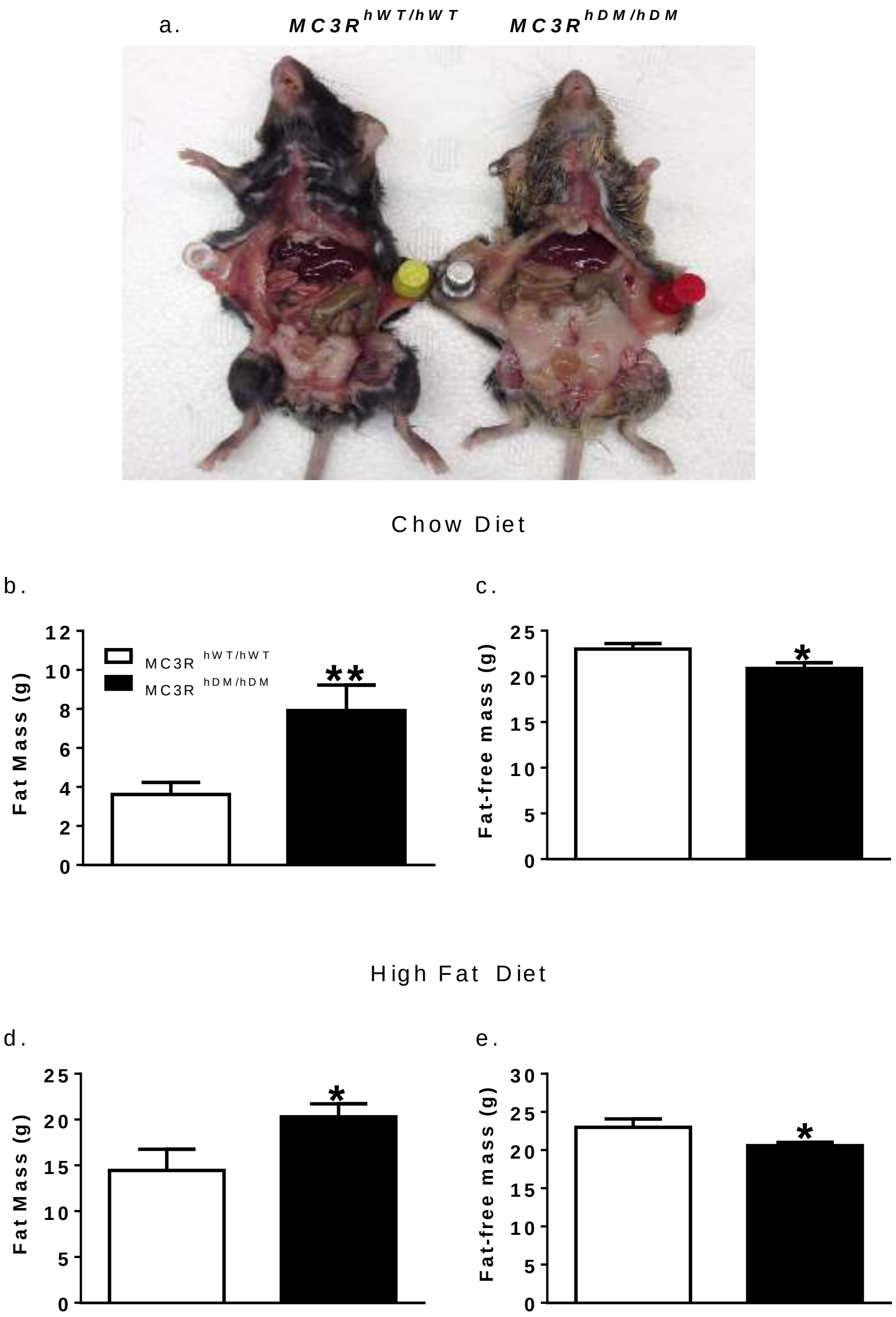

Supplementary Figure 4. Greater fat mass and reduced fat-free mass in male *MC3R*$^{hDM/hDM}$ mice. (a) Chow-fed male (3 month old) *MC3R*$^{hDM/hDM}$ mice demonstrate increased adiposity compared to *MC3R*$^{hWT/hWT}$. Male *MC3R*$^{hWT/hWT}$ (open bars) or *MC3R*$^{hDM/hDM}$ (closed bars) mice were fed (b-c) chow diet (n=10 *MC3R*$^{hWT/hWT}$; *MC3R*$^{hDM/hDM}$ n=9) or (d-e) high-fat diet (*MC3R*$^{hWT/hWT}$ n=7; *MC3R*$^{hDM/hDM}$ 8/group) for 2 months. Body fat (b, d) and fat-free mass (c, e) were measured by MRI at age 2-3 months for chow-fed mice and at 4-5 month for high-fat-fed mice. Data are represented as mean ± SEM. *P<0.05 and **P<0.01 *MC3R*$^{hDM/hDM}$ vs. *MC3R*$^{hWT/hWT}$ mice.

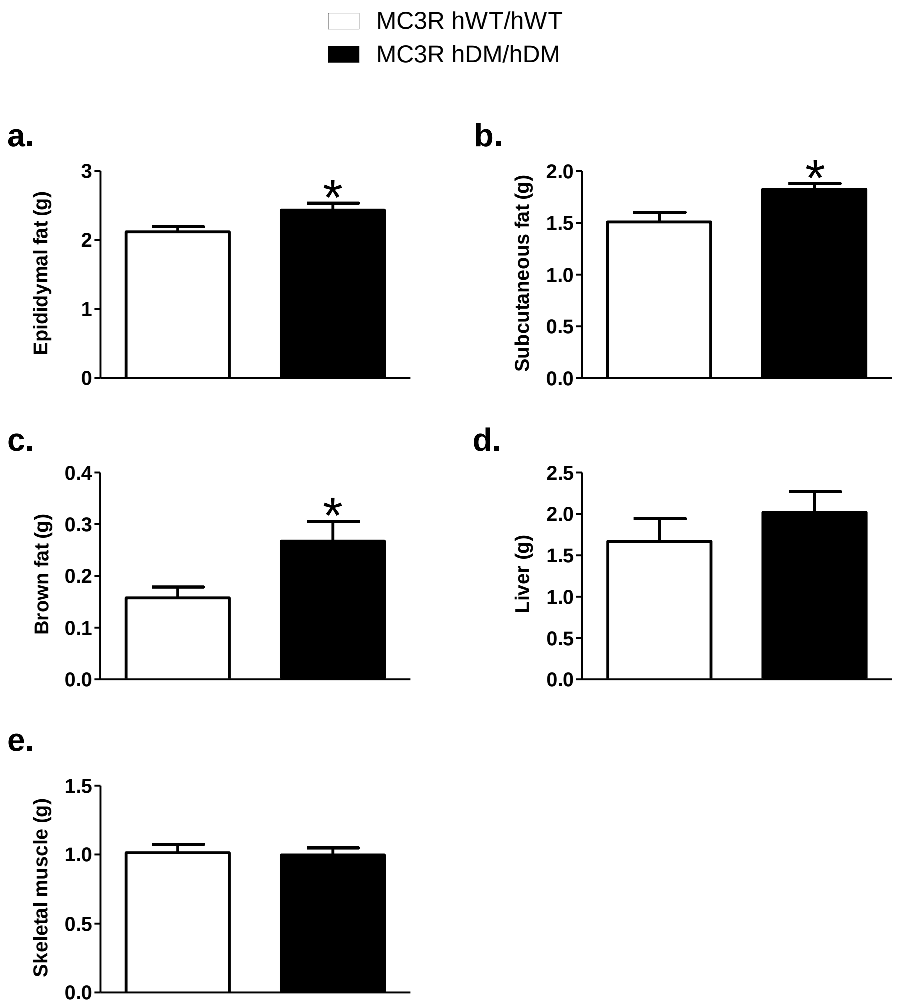

Supplementary Figure 5. Increased fat mass in *MC3R*$^{hDM/hDM}$ mice was not fat depot-specific. *MC3R*$^{hWT/hWT}$ (open bars) or *MC3R*$^{hDM/hDM}$ (closed bars) male mice were fed a high-fat diet (4/group) for 2 months. The weight of epididymal fat (a), subcutaneous flank fat (b), interscapular brown fat (c), liver (d), and skeletal muscle dissected from both legs (e) were measured after overnight fasting. Data are represented as mean ± SEM. $*P<0.05$ *MC3R*$^{hDM/hDM}$ vs. *MC3R*$^{hWT/hWT}$ mice. Similar results were found in female mice (data not shown).

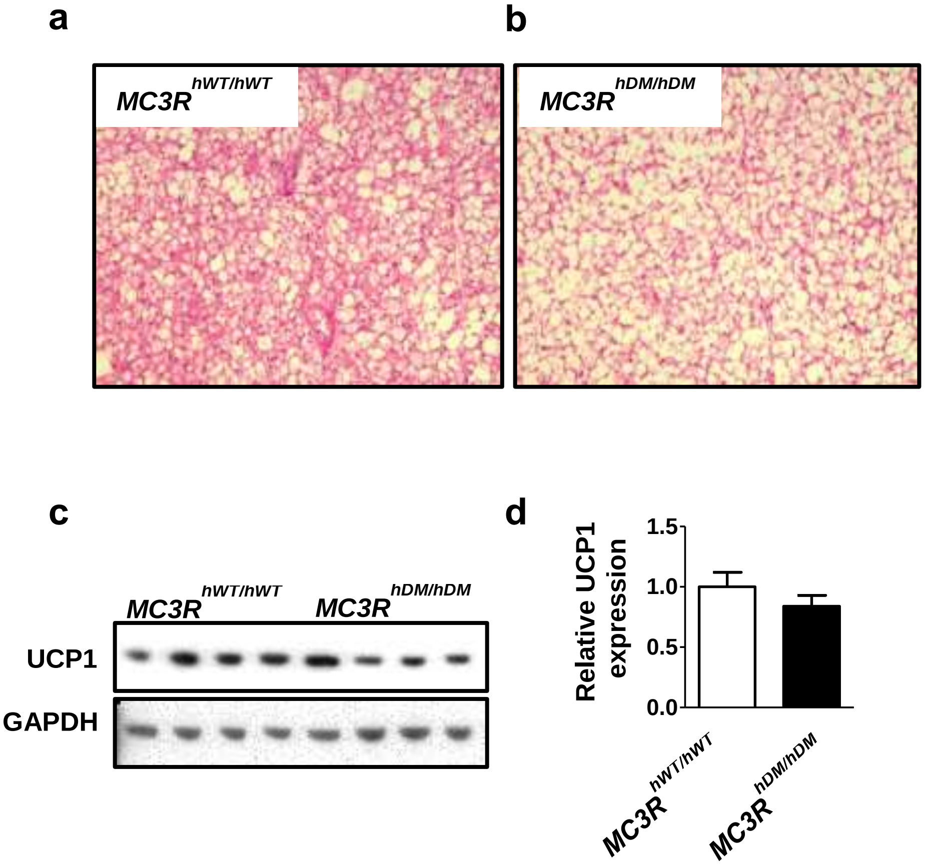

Supplementary Figure 6. Lipid accumulation is increased but UCP1 protein expression was not altered in brown adipose tissue of *MC3R*$^{hDM/hDM}$ mice. (a-b): Histology was performed from *MC3R*$^{hWT/hWT}$ (open bars) or *MC3R*$^{hDM/hDM}$ (closed bars) female mice fed a high-fat diet for 2 months(*MC3R*$^{hWT/hWT}$ n=6; *MC3R*$^{hDM/hDM}$ n=9). Brown adipose tissue was stained with hematoxylin and eosin (n=3/group, scale bar: 100μm). UCP1 protein expression was measured by western blotting (c) and semi-quantified (n=4/group) (d). Data are represented as mean ± SEM.

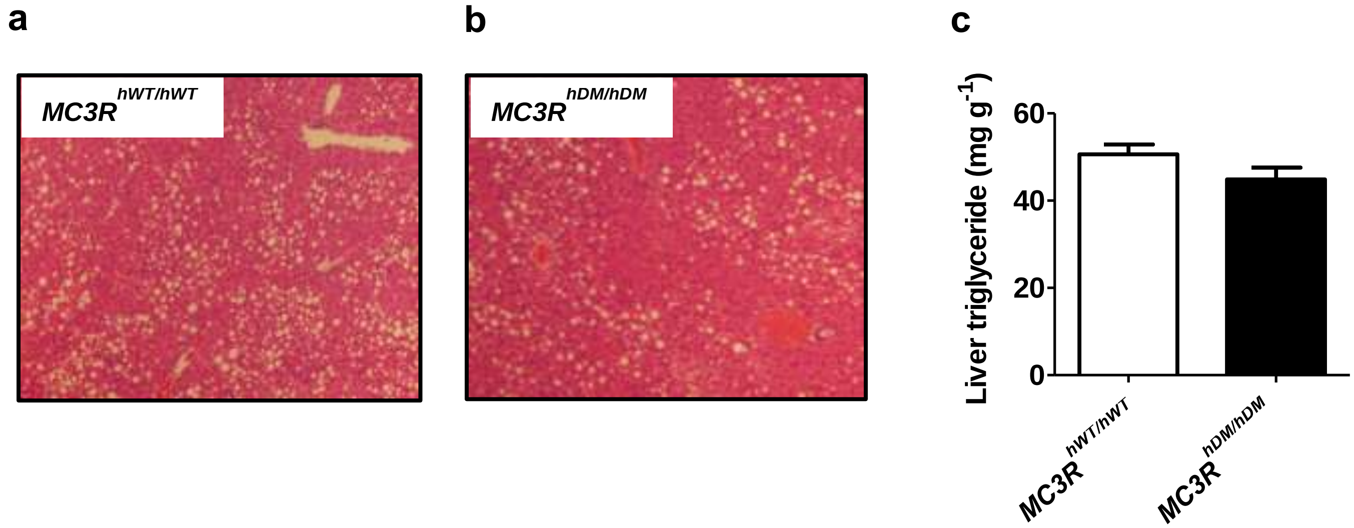

Supplementary Figure 7. **Liver triglyceride deposition was not increased in *MC3R*$^{hDM/hDM}$ mice.** *MC3R*$^{hWT/hWT}$ (open bars) or *MC3R*$^{hDM/hDM}$ (closed bars) female mice were fed a high-fat diet (*MC3R*$^{hWT/hWT}$ n=6; *MC3R*$^{hDM/hDM}$ n=9) for 2 months and studied after an overnight fast. Liver samples were stained with hematoxylin and eosin (Scale bar: 100µm) (a-b). Mean ± SEM tissue triglyceride concentration (c) was measured by an enzymatic assay.

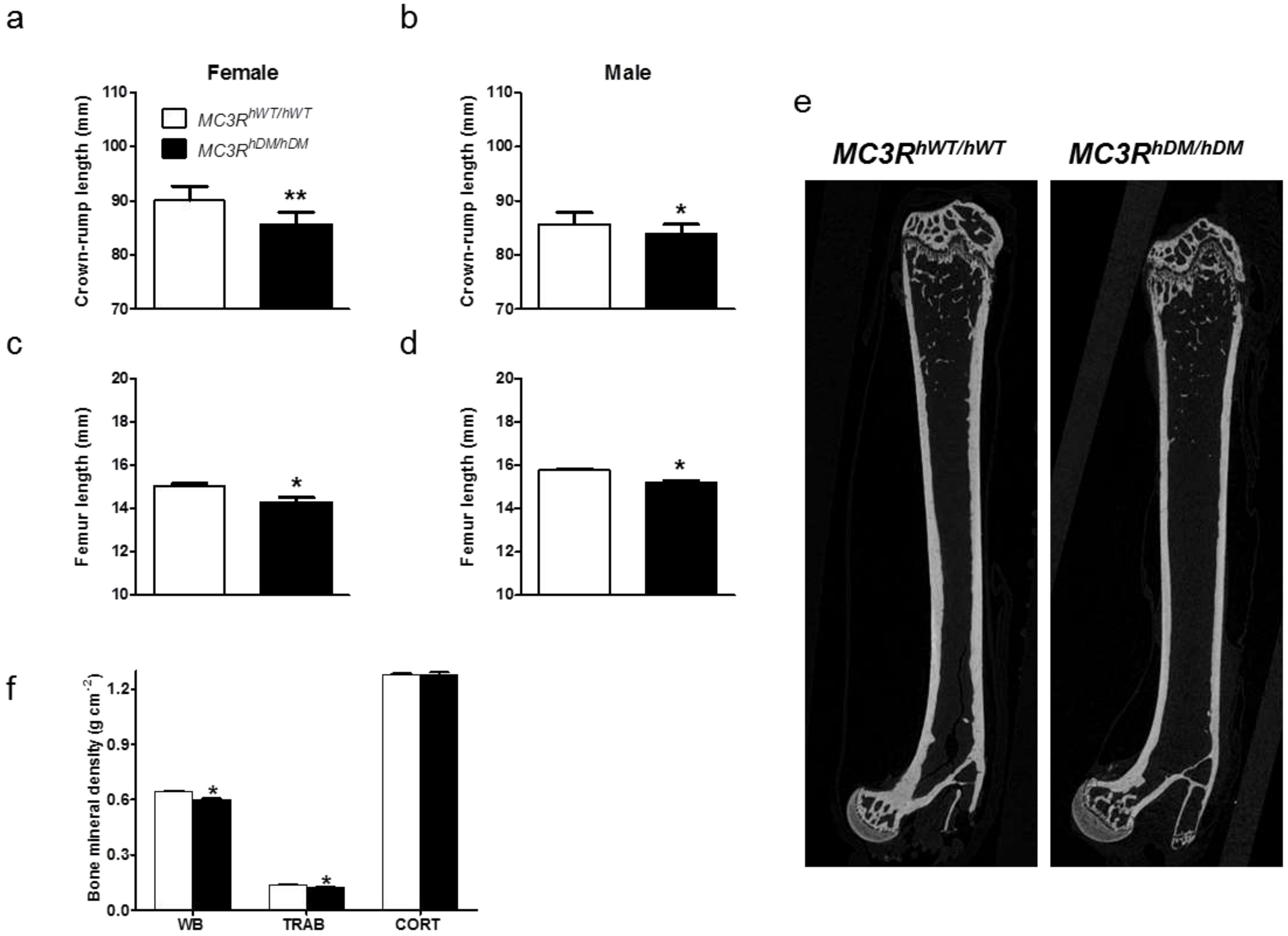

Supplementary Figure 8. Crown-Rump and Femur length were decreased in MC3R$^{hDM/hDM}$ compared to *MC3R$^{hWT/hWT}$* mice. (a, b) Crown-rump length was measured from male (*MC3R$^{hWT/hWT}$* n=9; *MC3R$^{hDM/hDM}$* n=12) and female (*MC3R$^{hWT/hWT}$* n=6; *MC3R$^{hDM/hDM}$* n=8) mice matched for age (10-12 weeks). (c, d) Femur length was obtained from micro-CT 3D reconstructed images with male (n=5/group) and female (n=6/group) mice at age 12 weeks. (e) Representative images of whole femurs are shown. (f) Bone Mineral Density (BMD) of female *MC3R$^{hWT/hWT}$* and *MC3R$^{hDM/hDM}$* mice. Data demonstrate reduced BMD of Whole Bone (WB) and Trabecular (TRAB) bone; however cortical BMD showed no difference in BMD compared to *MC3R$^{hWT/hWT}$* mice.

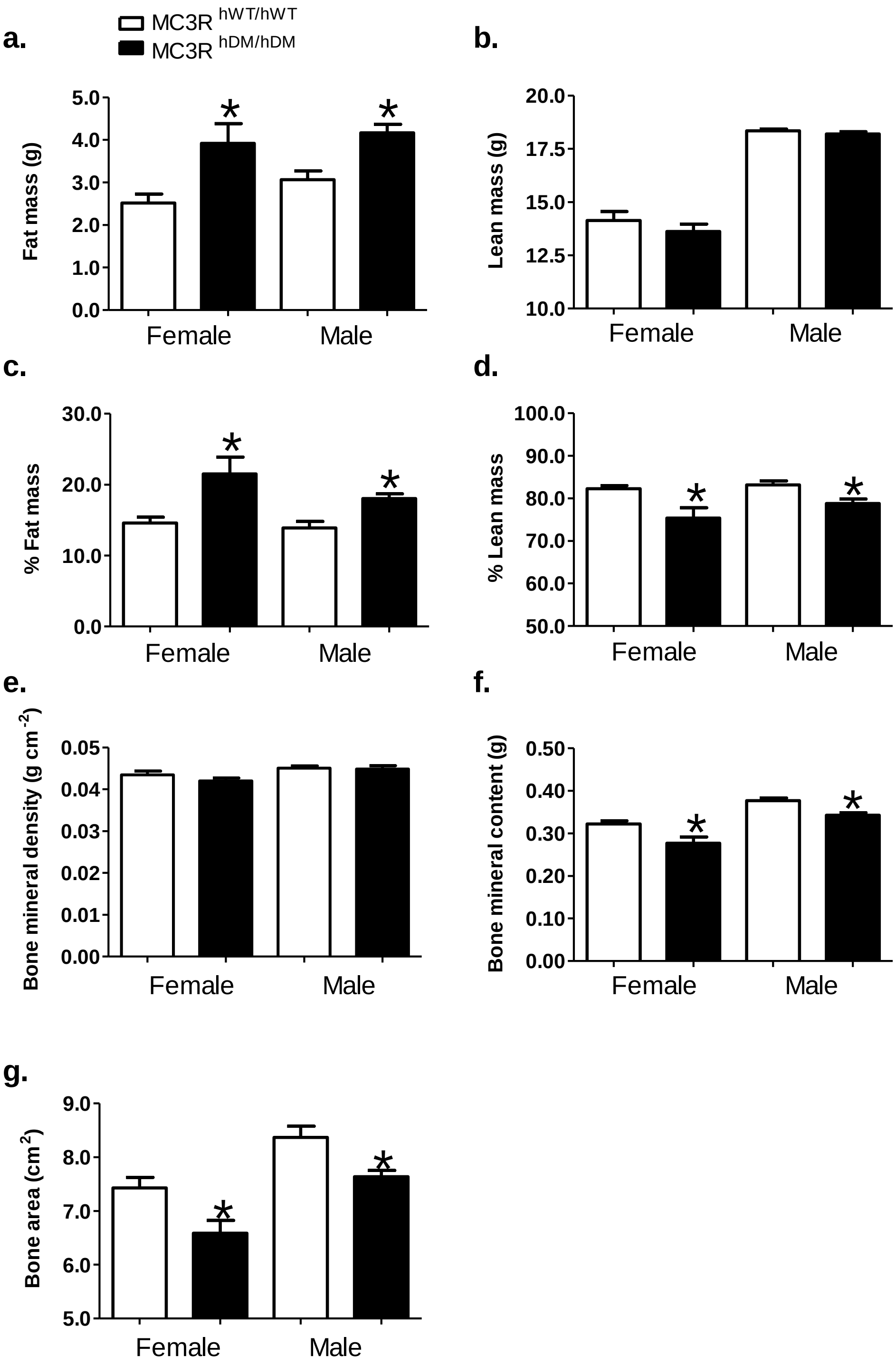

Supplementary Figure 9. Dual-Energy X-ray Absorptiometry in *MC3R*$^{hDM/hDM}$ and *MC3R*$^{hWT/hWT}$ mice. Chow-fed female and male *MC3R*$^{hWT/hWT}$ (open bars) or *MC3R*$^{hDM/hDM}$ (closed bars) mice (n= 5/group for female, n=3/group for male) were used for measuring (a) body fat, (b) lean mass, (c) percent fat mass, (d) percent lean mass, (e) bone mineral density, (f) bone mineral content, and (g) bone area by DEXA at age 8-9 weeks for female and age 9-10 weeks for male mice. Data are represented as mean ± SEM. *P<0.05 *MC3R*$^{hDM/hDM}$ vs. *MC3R*$^{hWT/hWT}$ mice.

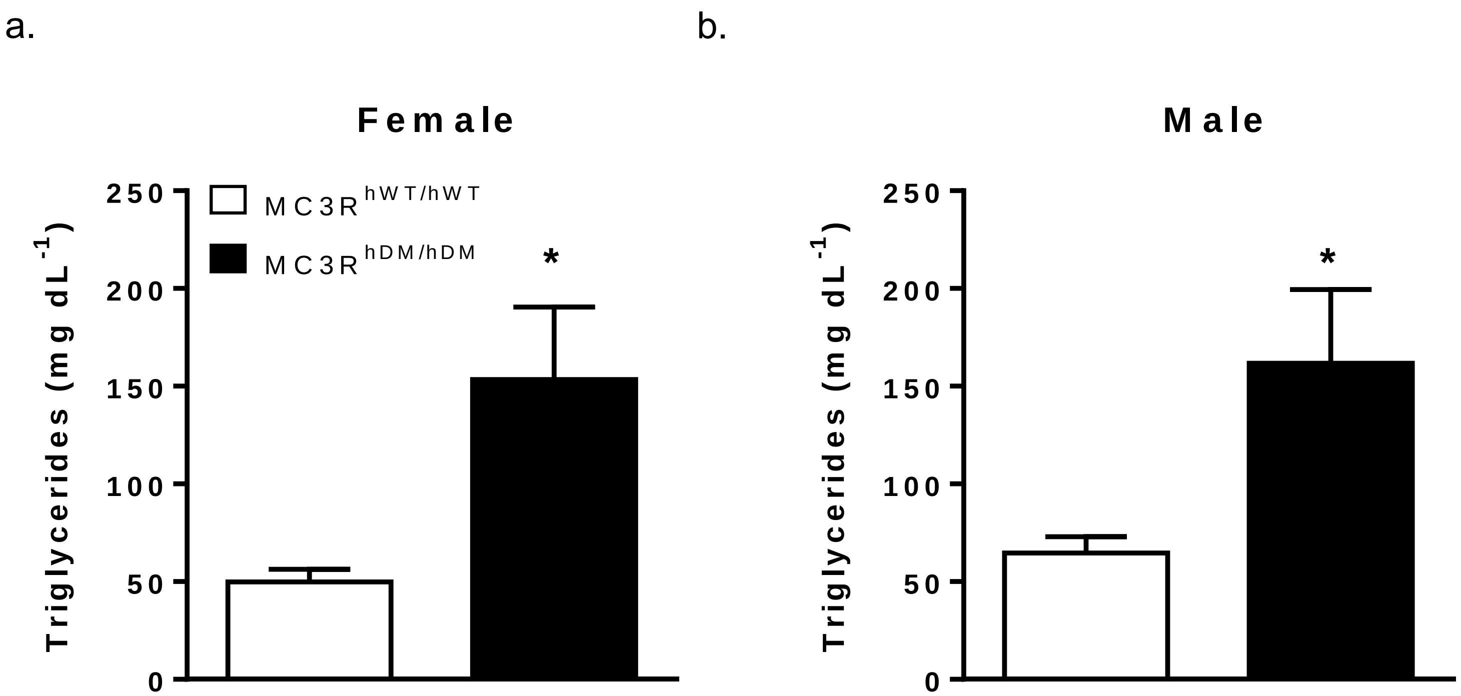

Supplementary Figure 10. Triglyceride content of bone marrow cells was significantly increased in MC3R$^{hDM/hDM}$ versus *MC3R*$^{hWT/hWT}$ mice. Bone marrow cells were removed from femurs of 3 month old, chow fed (a) female (n= *MC3R*$^{hWT/hWT}$ n=5; *MC3R*$^{hDM/hDM}$ n=4) and (b) male (n=5/group) MC3R$^{hWT/hWT}$ and MC3R$^{hDM/hDM}$ mice. Three independent measurements were performed in each experiment. Triglycerides were extracted and the concentrations measured by an enzymatic assay. See Methods for detailed procedures. *P<0.05 *MC3R*$^{hDM/hDM}$ vs. *MC3R*$^{hWT/hWT}$ mice.

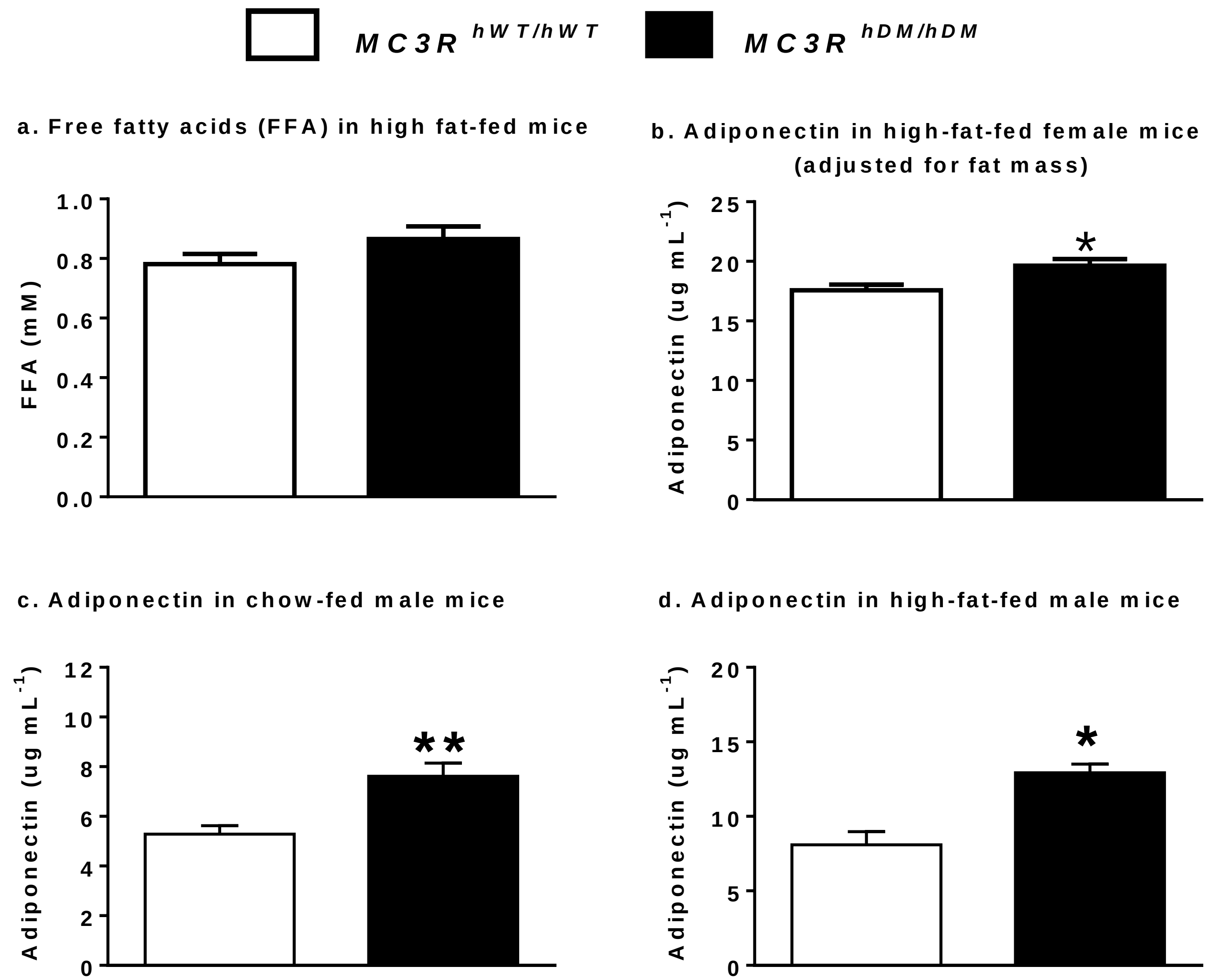

Supplementary Figure 11. Similar free fatty acids and greater serum adiponectin in *MC3R*$^{hDM/hDM}$ than in *MC3R*$^{hWT/hWT}$ mice. For measuring serum (a) free fatty acids (*MC3R*$^{hWT/hWT}$ n=21; *MC3R*$^{hDM/hDM}$ n=18) and (b) adiponectin (*MC3R*$^{hWT/hWT}$ n=7; *MC3R*$^{hDM/hDM}$ n=9) in female mice, blood was collected from tail vein of 5 month old mice fed a high fat diet after overnight fasting. Adiponectin concentration was adjusted for fat mass for female. For measuring (c) adiponectin in chow-fed (*MC3R*$^{hWT/hWT}$ n=10; *MC3R*$^{hDM/hDM}$ n=8) or (d) high fat-fed (*MC3R*$^{hWT/hWT}$ n=6; *MC3R*$^{hDM/hDM}$ n=8) male mice, blood was collected from tail vein of 9-10 week old male mice fed a chow diet in the fed state or 5 months old male mice fed a high fat diet for 10 weeks in the fasted state. Commercially available kits were used (see RESEARCH DESIGN AND METHODS). Data are represented as mean ± SEM. *P<0.05 vs. *MC3R*$^{hWT/hWT}$.

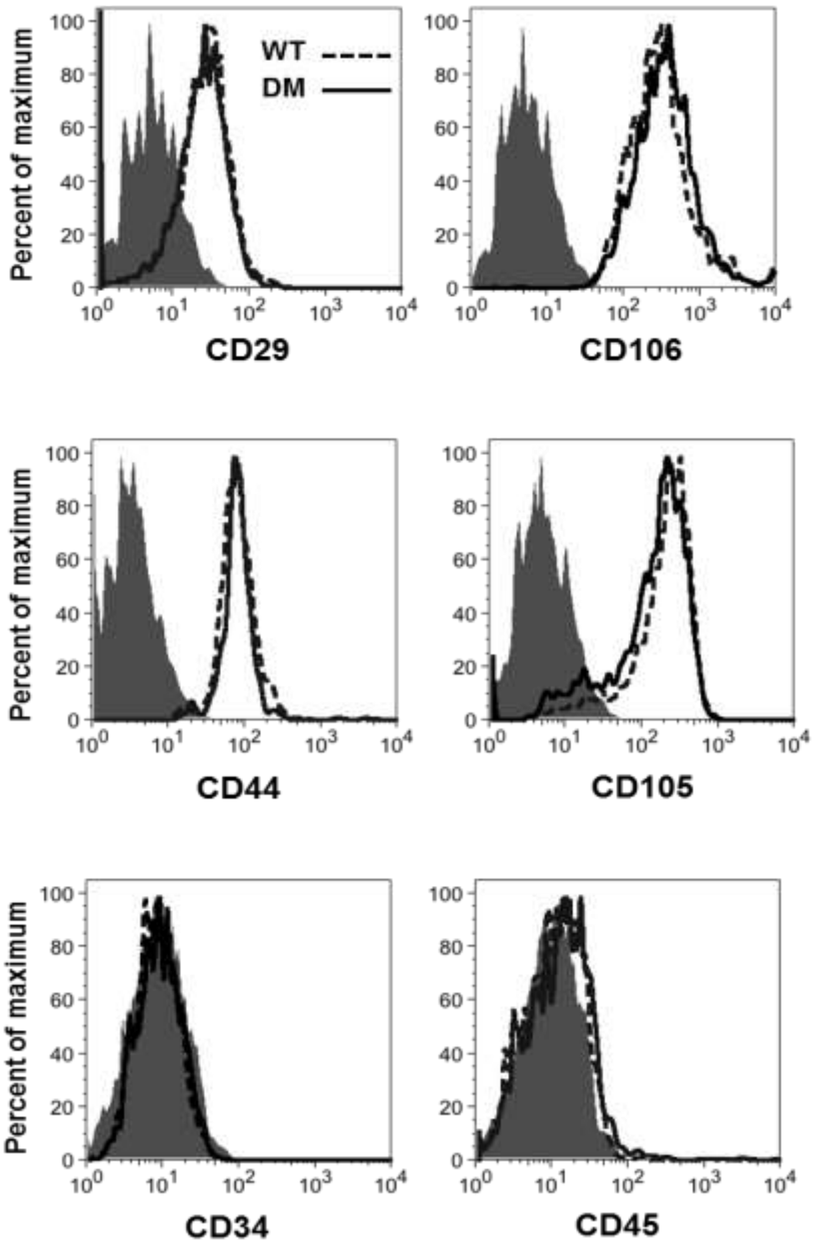

Supplementary Figure 12. Purity of mesenchymal stem cells (MSCs) isolated from compact bone of *MC3R*$^{hWT/hWT}$ and *MC3R*$^{hDM/hDM}$ mice. Tibiae and femurs from chow-fed female *MC3R*$^{hWT/hWT}$ (WT; dotted line) and *MC3R*$^{hDM/hDM}$ (DM, solid line) mice (n= 3/group) were used for isolating MSCs. Isolated MSCs were cultured in 25cm cell culture dishes for 4 passages (see method for detailed information). MSCs (4 passages) were analyzed by flow cytometry for purity. CD29 (a), CD106 (b), CD44 (c), and CD105 (d) were used to detect MSCs. CD34 (primitive hematopoietic progenitor and endothelial cell marker) and CD45 (pan-leukocyte marker) were used as negative MSC markers to check purity of MSCs. The dark grey image represents control MSCs without antibody treatment.

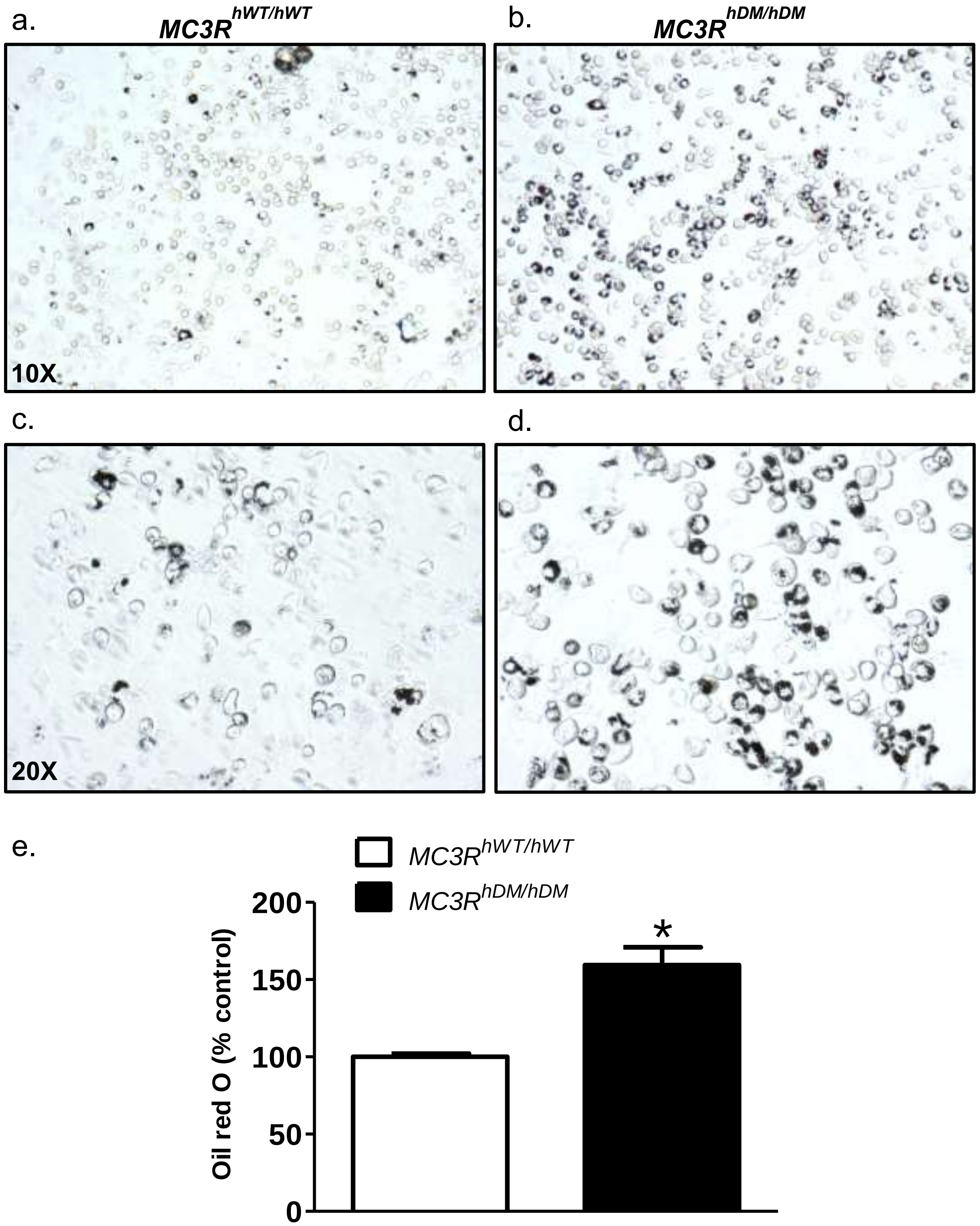

Supplementary Figure 13. Increased adipogenic capacity of preadipocytes isolated from *MC3R*$^{hDM/hDM}$ compared to *MC3R*$^{hWT/hWT}$ mice. SVF was isolated from epididymal adipose tissue of 4-5 month old female mice. Adipose tissues from two mice were combined for cultures and three independent experiments were performed (n=6/group). Cells from the SVF were differentiated into adipocytes using adipogenic stimulation medium as per Methods. Oil red O staining was performed. (a-d) Microscopic images of Oil-red O stained adipocytes (Scale bar:100μm) (e) Oil-red O was extracted from adipocytes and quantified at 520nm.

Supplementary Table 1

A: Chow diet condition*				
	Energy intake (Kcal/mouse)	Energy intake (Kcal/mouse adj. for fat-free mass)	Energy intake (Kcal/mouse adj. for fat-free & fat mass)	Energy intake (Kcal/mouse adj. for body weight)
MC3R $^{hWT/hWT}$ (n=5)	10.14 ± 0.29	10.15 ± 0.31	10.60 ± 0.36	10.39 ± 0.32
MC3R $^{hDM/hDM}$ (n=5)	11.23 ± 0.29	11.23 ± 0.31	10.77 ± 0.36	10.98 ± 0.32
p-value	**0.028**	**0.044**	0.794	0.301
B: High-Fat diet condition*				
	Energy intake (Kcal/mouse)	Energy intake (Kcal/mouse adj. for fat-free mass)	Energy intake (Kcal/mouse adj. for fat-free & fat mass)	Energy intake (Kcal/mouse adj. for body weight)
MC3R $^{hWT/hWT}$ (n=5)	13.33. ± 0.45	13.47 ± 0.47	14.17 ± 0.64	13.29 ± 0.49
MC3R $^{hDM/hDM}$ (n=5)	15.25 ± 0.45	15.11 ± 0.47	14.41 ± 0.64	15.29 ± 0.49
p-value	**0.017**	**0.048**	0.837	**0.025**

Supplementary Table 1: Energy Intake. A: Energy intake during chow diet condition. Daily energy intake for chow-fed (n=5/group) female *MC3R*$^{hWT/hWT}$ and *MC3R*$^{hDM/hDM}$ mice was measured for 2 weeks and adjusted for multiple factors. The adjusted energy intake values shown in the table are estimated marginal means from analysis of covariance. **B: Energy intake during High-Fat diet condition.** Daily energy intake for 45% high-fat-fed female *MC3R*$^{hWT/hWT}$ and *MC3R*$^{hDM/hDM}$ mice (n=5/group) was measured for 2 weeks and adjusted for multiple factors. The adjusted energy intake values shown in the table are estimated marginal means from analysis of covariance.

*Mean ± SEM. Adj. - Adjusted by ANCOVA for listed covariates. Estimated marginal means are shown.

Supplementary Table 2

A: Chow-fed condition*								
Group	Body Weight (g)	TEE (Kcal/mouse)	TEE (Kcal/mouse adj. for fat-free mass)	TEE (Kcal/mouse adj. for fat-free & fat mass)	TEE (Kcal/mouse adj. for body weight)	T-activity (Beam breaks·min^{-1})	A-activity (Beam breaks·min^{-1})	T-RER ($VCO_2 \cdot VO_2^{-1}$)
MC3R$^{hWT/hWT}$ (22°C)	22.86 ± 0.66	12.46 ± 0.43	12.41±0.49	12.21±0.86	12.45 ± 0.6	567±105	308±86	0.93±0.01
MC3R$^{hDM/hDM}$ (22°C	27.02 ± 1.07	11.77 ± 0.48	11.82±0.49	12.02±0.86	11.78 ± 0.6	740±178	397±128	0.87±0.02
p-value	**0.008**	0.303	0.434	0.904	0.513	0.425	0.578	**0.035**
MC3R$^{hWT/hWT}$ (30°C	23.05 ± 0.73	7.8 ± 0.3	7.74±0.28	7.84±0.49	8.0 ± 0.34	619±115	356±90	0.93±0.01
MC3R$^{hDM/hDM}$ (30°C	25.08 ± 0.98	6.91 ± 0.21	6.99±0.28	6.89±0.49	6.72 ± 0.34	641±144	347±102	0.92±0.02
p-value	0.1	**0.0375**	0.099	0.319	**0.048**	0.90	0.95	0.56

B. High-fat-fed condition*								
Group	Body Weight (g)	TEE (Kcal/mouse)	TEE (Kcal/mouse adj. for fat-free mass)	TEE (Kcal/mouse adj. for fat-free & fat mass)	TEE (Kcal/mouse adj. for body weight)	T-activity (Beam breaks·min^{-1})	A-activity (Beam breaks·min^{-1})	T-RER ($VCO_2 \cdot VO_2^{-1}$)
MC3R$^{hWT/hWT}$ (22°C)	22.94 ± 0.37	11.85±0.20	11.58±0.57	11.42±0.54	11.88±0.26	394±59	168±33	0.84±0.01
MC3R$^{hDM/hDM}$ (22°C)	22.54 ± 0.79	11.46±0.25	11.67±0.43	11.76±0.4	11.44±0.22	513±45	223±29	0.84±0.01
p-value	0.69	0.27	0.946	0.705	0.222	0.13	0.24	0.69
MC3R$^{hWT/hWT}$ (30°C)	23.98 ± 0.65	7.34±0.15	6.92±0.4	6.86±0.41	7.35±0.2	407±65	190±39	0.85±0.02
MC3R$^{hDM/hDM}$ (30°C)	23.81 ± 0.10	6.89±0.18	7.19±0.3	7.24±0.3	6.89±0.17	475±58	219±37	0.9±0.02
p-value	0.9	0.1	0.688	0.418	0.109	0.46	0.61	0.08

Supplementary Table 2: Energy Expenditure related parameters. A: Energy expenditure-related parameters in chow-fed condition. Total energy expenditure and activity of chow-fed (n=6/group) female mice at 22 °*C and* 30 °*C* were measured over a 24hr period by indirect calorimetry. The average of 24hr energy expenditure was adjusted for multiple factors by analysis of covariance. Respiratory exchange ratio (VCO_2 /VO_2) values were calculated based on VCO_2 and VO_2 values obtained during indirect calorimetry. The adjusted TEE values shown in the table are estimated marginal means from analysis of covariance. **B: Energy expenditure-related parameters in high-fat-fed condition.** Total energy expenditure and activity of high-fat-fed (n=5-7/group) female mice at 22 °*C and* 30 °*C* were measured over a 24hr period by indirect calorimetry. The average of 24hr energy expenditure was adjusted for multiple factors by analysis of covariance. Respiratory exchange ratio (VCO_2 /VO_2) values were calculated based on VCO_2 and VO_2 values obtained during indirect calorimetry. The adjusted TEE values shown in the table are estimated marginal means from analysis of covariance. Abbreviations: TEE, total energy expenditure, T-activity, total activity, A-activity, ambulatory activity, T-RER, total respiratory exchange ratio, Adj, adjusted by ANCOVA for listed covariates. Estimated marginal means are shown.
*Mean ± SEM

Supplementary Table 3. Mouse primer sequences

	Forward	Reverse
F4/80	GGTGGGACCACAGAGAGTTG	CCTGGACGAATCCTGTGAAG
Chi3l3	GACCATGGCACTGAACGAG	CAAGAACACTGAGCTAAAAACTCTCCTG
CD68	CGCCATGAATGTCCACTG	GACCTACATCAGAGCCCGAGT
MCP1	CTTCCGGACGTGAATCTTCT	CCATCAGTCCTCAGGTATTGG
IL-6	CCAGGTAGCTATGGTACTCCAGAA	GCTACCAAACTGGATATAATCAGGA
β-actin	GGGGTGTTGAAGGTCTCAAA	CTGAACCCTAAGGCCAACC
FAS	CCCTTGATGAAGAGGGATCA	ACTCCACAGGTGGGAACAAG
Adiponectin	CGAATGGGTACATTGGGAAC	AAAGGAGAGCCTGGAGAAGC
PPARγ	GAAAGACAACGGACAAATCACC	GGGGGTGATATGTTTGAACTTG
C/EBPα	TGGACAAGAACAGCAACGAG	TCACTGGTCAACTCCAGCAC

Supplementary Table 4. Demographic and Body Composition data for human subjects used to measure plasma adiponectin

	Human MC3R WT (n=13)	Human MC3R DM (n=13)	p-value
Age* (y)	14.5 ± 0.34	15.1 ± 0.39	0.19
Race	100% African American	100% African American	1.0
Sex	F=7 and M=6	F=10 and M=3	0.20
Weight* (kg)	104.9 ± 6.4	112.2 ± 6.9	0.45
Height* (cm)	161.3 ± 3.3	164.4 ± 2.6	0.47
BMI* (kg m^{-2})	41.5 ± 3.7	41.4 ± 2.2	0.98
BMIz score*	2.43 ± 0.22	2.39± 0.22	0.91
Fat mass* (%)	40.1±2.9	43.5±2.2	0.36

*Mean ± SEM. Fat mass measured by dual-energy X-ray absorptiometry.
